# Expanding a precision medicine platform for malignant peripheral nerve sheath tumors: New patient‐derived orthotopic xenografts, cell lines and tumor entities

**DOI:** 10.1002/1878-0261.13534

**Published:** 2023-10-20

**Authors:** Edgar Creus‐Bachiller, Juana Fernández‐Rodríguez, Miriam Magallón‐Lorenz, Sara Ortega‐Bertran, Susana Navas‐Rutete, Cleofe Romagosa, Tulio M. Silva, Maria Pané, Anna Estival, Diana Perez Sidelnikova, Mireia Morell, Helena Mazuelas, Meritxell Carrió, Tereza Lausová, David Reuss, Bernat Gel, Alberto Villanueva, Eduard Serra, Conxi Lázaro

**Affiliations:** ^1^ Hereditary Cancer Program Catalan Institute of Oncology, ICO‐IDIBELL, Hospitalet de Llobregat Barcelona Spain; ^2^ Program in Molecular Mechanisms and Experimental Therapy in Oncology (Oncobell), IDIBELL, Hospitalet de Llobregat Barcelona Spain; ^3^ Mouse Lab, IDIBELL, Hospitalet de Llobregat Barcelona Spain; ^4^ Centro de Investigación Biomédica en Red de Cáncer (CIBERONC) Madrid Spain; ^5^ Hereditary Cancer Group, Germans Trias i Pujol Research Institute (IGTP) Barcelona Spain; ^6^ Department of Pathology Hospital Vall d'Hebron Barcelona Spain; ^7^ Department of Pathology HUB‐IDIBELL, L'Hospitalet de Llobregat Barcelona Spain; ^8^ Department of Medical Oncology Catalan Institute of Oncology Barcelona Spain; ^9^ Plastic Surgery Service HUB‐IDIBELL, L'Hospitalet de Llobregat Barcelona Spain; ^10^ Department of Neuropathology Institute of Pathology, Heidelberg University Hospital Heidelberg Germany; ^11^ Clinical Cooperation Unit Neuropathology German Cancer Research Center (DKFZ), German Consortium for Translational Cancer Research (DKTK) Heidelberg Germany; ^12^ Procure Program Catalan Institute of Oncology Barcelona Spain

**Keywords:** cellular models, MPNST, NF1, PDOX, treatment response, tumor entities

## Abstract

Malignant peripheral nerve sheath tumors (MPNSTs) are aggressive soft‐tissue sarcomas with a poor survival rate, presenting either sporadically or in the context of neurofibromatosis type 1 (NF1). The histological diagnosis of MPNSTs can be challenging, with different tumors exhibiting great histological and marker expression overlap. This heterogeneity could be partly responsible for the observed disparity in treatment response due to the inherent diversity of the preclinical models used. For several years, our group has been generating a large patient‐derived orthotopic xenograft (PDOX) MPNST platform for identifying new precision medicine treatments. Herein, we describe the expansion of this platform using six primary tumors clinically diagnosed as MPNSTs, from which we obtained six additional PDOX mouse models and three cell lines, thus generating three pairs of *in vitro–in vivo* models. We extensively characterized these tumors and derived preclinical models, including genomic, epigenomic, and histological analyses. Tumors were reclassified after these analyses: three remained as MPNSTs (two being classic MPNSTs), one was a melanoma, another was a neurotrophic tyrosine receptor kinase (*NTRK*)‐rearranged spindle cell neoplasm, and, finally, the last was an unclassifiable tumor bearing neurofibromin‐2 (*NF2*) inactivation, a neuroblastoma RAS viral oncogene homolog (*NRAS*) oncogenic mutation, and a SWI/SNF‐related matrix‐associated actin‐dependent regulator of chromatin (*SMARCA4*) heterozygous truncated variant. New cell lines and PDOXs faithfully recapitulated histology, marker expression, and genomic characteristics of the primary tumors. The diversity in tumor identity and their specific associated genomic alterations impacted treatment responses obtained when we used the new cell lines for testing compounds against known altered pathways in MPNSTs. In summary, we present here an extension of our MPNST precision medicine platform, with new PDOXs and cell lines, including tumor entities confounded as MPNSTs in a real clinical scenario. This platform may constitute a useful tool for obtaining correct preclinical information to guide MPNST clinical trials.

## Graphical abstract

Malignant peripheral nerve sheath tumors (MPNSTs) are aggressive soft‐tissue sarcomas with a poor patient survival rate. New treatment options need to be developed using genuine MPNST *in vitro*/*in vivo* models. We present here an extension of our MPNST precision medicine platform, with new PDOXs and cell lines, including tumor entities confounded as MPNSTs in a real clinical scenario. This platform constitutes a useful tool for obtaining reliable preclinical information to guide MPNST clinical trials.

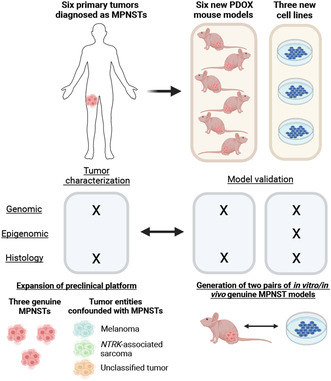

**Abbreviations**
ANNUBP: Atypical neurofibromatous with unknown biological potential
BAF: Biallelic frequency
CI: Combination Index
CN: Copy number
GAP: Genome Alteration Print
IC50: Half‐maximal inhibitory dilution
ICGC: International Cancer Genome Consortium
IGV: Integrative Genome Viewer
LOH: Loss of heterozygosity
LRR: Log R ratio
MPNST: Malignant peripheral nerve sheath tumor
NF1: Neurofibromatosis type 1
PDOX: Patient‐derived orthotopic xenograft
PDT: Population doubling time
PRC2: Polycomb repressive complex 2
SNV: Single nucleotide variant
SV: Structural variant
TSG: Tumor suppressor gene
UMAP: Uniform Manifold Approximation and Projection
VAF: Variant allele frequency
WES: Whole exome sequencing
WGS: Whole genome sequencing
WT: wild‐type

## 1 Introduction

Malignant peripheral nerve sheath tumors (MPNSTs) account for about 3–10% of all soft‐tissue sarcomas [1]; half of them occur in patients with neurofibromatosis type 1 (NF1), an autosomal dominant genetic disorder with an incidence at birth of 1 : 2000–1 : 3000 [2, 3]. The lifetime risk of developing an MPNST in NF1 patients is around 8–15% [4, 5], constituting the leading cause of mortality in these patients [4, 6]. In the NF1 clinical context, MPNSTs usually arise from preexisting benign plexiform neurofibromas (pNF), which can undergo premalignant transformation into atypical neurofibromas (ANNUBPs) before MPNST generation. ANNUBPs, in addition to *NF1* loss, harbor *CDKN2A* inactivation as a common genomic loss in the progression toward MPNSTs [7, 8]. Malignant peripheral nerve sheath tumor cells contain highly rearranged and hyperploid genomes with a low mutation burden and few recurrent alterations [9]. A core MPNST tumor suppressor gene (TSG) mutational pattern consists of the recurrent inactivation of *NF1*, *CDKN2A*, and components of the polycomb repressive complex 2 (PRC2; *SUZ12* and *EED*); less frequently, *TP53* is also inactivated [10, 11, 12]. Interestingly, some drugs can target these pathways, such as MEK inhibitors (*NF1* loss), CDK4/CDK6 inhibitors (loss of *CDKN2A*), and BRD4 inhibitors (PRC2 loss of function), and some have been tested in preclinical [13, 14] or clinical [15, 16, 17] contexts. However, clinical trials of typical cytotoxic drugs have shown response rates ranging from 18 to 44%, indicating that drug combinations will be required for efficient treatment [18]. Doxorubicin and ifosfamide have been used as the standard chemotherapy regimen for MPNSTs; however, a 10‐year institutional review found no correlation between chemotherapy and patient survival [19]. Currently, complete surgical excision with clear margins is the standard treatment option for local MPNST disease [20, 21]; nevertheless, its success is limited by tumor infiltration, resulting in a high relapse rate [21]. In addition, the diagnosis of MPNSTs may be challenging, especially in the sporadic context, which may contribute to low efficacy of MPNST treatments. Nowadays, specific histological criteria for MPNST diagnosis are lacking [22, 23], and other tumor entities share histological characteristics with MPNSTs. The more usual MPNST histology includes the presence of spindle cells with a fascicular growth pattern and areas with high hypercellularity, sometimes called a ‘classic’ MPNST [24]. However, in many cases, MPNST histology may differ from this usual pattern.

*In vitro* and *in vivo* models are paramount to studying MPNST biology and testing new therapeutic approaches. At least 44 NF1 or sporadic MPNST cell lines from primary tumors, metastases, or mice tumors have been described (Cellosaurus version 45, updated in March 2023) [25, 26]. Several *in vivo* tumor models have been developed to study MPNSTs, including xenograft models of patient‐derived cells injected subcutaneously or orthotopically [25], genetically engineered mouse models (reviewed in [27]), and patient‐derived xenografts [28, 29, 30, 31]. Our laboratory previously reported the establishment and validation of four MPNST patient‐derived orthotopic xenograft (PDOX) mouse models [32]. We also demonstrated that PDOX mouse models closely resemble primary tumors at different levels, histologically and molecularly [32].

Over several years, our group has collected a total of 43 primary, relapsed, and metastatic tumors clinically diagnosed as MPNSTs from NF1 and sporadic patients and have generated PDOX models from most of them for precision medicine preclinical studies and the discovery of new therapeutic treatments [33, 34]. In this work, we enlarge our preclinical platform by characterizing, at the molecular and histological level, six primary tumors diagnosed as MPNSTs by clinical pathology. Furthermore, we describe the establishment of six PDOX models and three cell lines directly derived from primary tumors, generating three cell line‐PDOX model pairs from the same tumors. Finally, the established cell lines were used to test different known MPNST drugs, evidencing that both genomic status and misidentification of tumor entities are at least partially responsible for the observed heterogeneity in MPNST treatment response.

## 2 Materials and methods

### 2.1 Patients, animal, and cell models

#### 2.1.1 Primary tumor acquisition and processing

Six primary tumors from different unrelated patients were identified and removed from January 2011 to March 2016 at different hospitals from the Barcelona area (Bellvitge, Vall Hebron, and Germans Trias i Pujol). Clinical data from the patients are summarized in Table S1. Five of the patients did not receive any treatment before surgery. Only one patient (SP‐06) received neoadjuvant radiotherapy. After surgery, a piece of each tumor was stored in DMEM culture medium supplemented with 10% fetal bovine serum (FBS; Gibco, Waltham, MA, USA) at room temperature (RT) before being sent to our laboratory. Small pieces of each tumor were directly frozen in liquid nitrogen for DNA, RNA, and protein extraction; other fragments were frozen in FBS with 10% DMSO for cell culture establishment and mouse engraftment. Written informed consent was obtained from all subjects, and the study received IDIBELL IRB (#PR213/13) approval. The experimental protocols followed the Declaration of Helsinki.

#### 2.1.2 Animal care conditions

Six‐week‐old male Athymic Nude‐Foxn1^nu^ (Envigo, Indianápolis, IN, USA) mice weighing 18 to 22 g were used in this study. Animals were housed in a sterile environment, in cages with autoclaved bedding, food, and water. The mice were maintained on a daily 12‐h light/12‐h dark cycle.

#### 2.1.3 Human tumor engraftment for PDOX generation and perpetuation

Fresh surgical specimens were implanted in athymic nude mice, as described previously [32]. Briefly, fresh surgical specimens were minced into small fragments 2–3 mm^3^ in size, grafted close to the sciatic nerve, and grown surrounding the epineurium. The MPNST‐PDOX procedure was approved by the campus Animal Ethics Committee and complied with AAALAC (Association for Assessment and Accreditation of Laboratory Animal Care International) procedures.

#### 2.1.4 Establishment of cell lines from primary human tumors

Fresh tumors were minced into small fragments and digested with 100 U·mL^−1^ collagenase (C0130; Sigma‐Aldrich, Burlington, MA, USA) and 1 U·mL^−1^ dispase (LS02100; Worthington Corporations, Lakewood, NJ, USA) in DMEM medium supplemented with 10% FBS and 100 μg·mL^−1^ Penicillin/Streptomycin (BioWest, Nuaillé, France). After 18 h of incubation, digested tissue was filtered through a 40 μm filter to seed single cells in 6‐well plates. Cell lines were initially maintained for 10 passages at 37 °C and 10% CO_2_. Subsequently, cells were cultured at 5% CO_2_. In this work, the following established cell lines were also used: NF1‐derived 88‐14 (RRID: CVCL_8916) [35] and S462 (RRID: CVCL_1Y70) [36], and sporadic STS‐26T (RRID: CVCL_8917) [37]. All details regarding these three cell lines, as well as the laboratories originating and providing these cell lines, are described in Magallón‐Lorenz et al. [38]. Cell lines were validated as *Mycoplasma* negative and were retested every 2 months. Cell lines have been authenticated in the past 3 years by performing short tandem repeat (STR) profile authentication.

### 2.2 Tumor‐derived cell lines characterization

#### 2.2.1 Morphological analysis

Cells were plated in 10 cm plates and grown to 30% or 90% confluency to observe their morphology at low and high confluence, respectively, using a Leica DM IL LED optical microscope through Leica Microsystems' contrast phase mode (Leica Biosystems, Deer Park, IL, USA).

#### 2.2.2 STR authentication

DNA fingerprints were obtained using the AmpFLSTR Identifiler Plus PCR Amplification kit (Applied Biosystems, Waltham, MA, USA), according to the manufacturer's protocol. The combination of markers used is consistent with worldwide recommendations for identity testing. The kit amplifies 15 tetranucleotide STR loci and the gender‐determining marker, amelogenin, in a single PCR amplification.

#### 2.2.3 Fluorescence immunostaining

Cells were plated in 12‐well Corning^®^ (Corning, NY, USA) plates using coverslips (12 mm Ø) and fixed for 15 min in 4% paraformaldehyde when highly confluent. Then, cells were permeabilized in PBS 1x‐0.1% Triton and blocked using PBS 1x‐10% Goat serum for 30 min. Primary antibodies SOX9 (1 : 100, ab76997; Abcam, Cambridge, UK), smooth muscle actin (SMA, 1 : 100, RB‐9010‐R7; ThermoFisher Scientific, Waltham, MA, USA), EGFR (1 : 50, ab32562; Abcam), p75 (1 : 100, AB‐N07; ATSbio, Carlsbad, CA), and S100B (1 : 1000, Z031129; Dako, Glostrup, Denmark) were diluted in PBS‐1% Goat serum and incubated overnight at 4 °C. Secondary antibodies Alexa Fluor 488 goat anti‐mouse (1 : 1000, A11029; Invitrogen, Waltham, MA, USA), Alexa Fluor 488 donkey anti‐rabbit (1 : 1000, 711‐545‐152; Jackson ImmunoResearch, Philadelphia, PA, USA), and Alexa Fluor 568 goat anti‐rabbit (1 : 1000, A11036; Invitrogen) were diluted in PBS‐10% Goat serum and incubated for 1 h at RT. After washing three times with PBS 1x, DAPI diluted in PBS (1 : 1000, 62248; ThermoFisher Scientific) was added for 10 min and then washed three times, and finally, coverslips were mounted in Immu‐Mount (9990402; ThermoFisher Scientific). Images were acquired using a Nikon Eclipse 80i fluorescence microscope with nis‐Elements Microscope Imaging Software and analyzed using imagej fiji software (Lexington, KY, USA).

#### 2.2.4 Cell cycle

A total of 2.5 × 10^5^ cells from a 50–60% confluent plate were fixed using 70% cold‐ethanol and dyed with a mixture of PBS‐1% FBS, propidium iodide (0.0625 mg·mL^−1^; Sigma‐Aldrich), and RNAse A (10 μg·mL^−1^; Sigma‐Aldrich) for 30–45 min at 37 °C. Samples were analyzed via a facscanto II (BD Bioscience, Franklin Lakes, NJ, USA) flow cytometer. Each cell line was analyzed in duplicate.

#### 2.2.5 Growth kinetics and migration properties

##### 2.2.5.1 Population doubling time

Population doubling times (PDTs) of cell lines were estimated using two different methodologies: dyeing cells with Trypan Blue (Sigma‐Aldrich) to count living cells using an optical microscope or by using a colorimetric cell viability assay [3‐(4,5‐dimethylthiazol‐2‐yl)‐2,5‐diphenyl‐tetrazolium bromide] (MTT). In the first approach, cell lines were seeded in triplicate in 6‐well plates to reach 100% confluence after 7–8 days of culture. Living cells were counted every 24 h using a Fast Reader 102^®^ (Biosigma, Cona, Italy). Population doubling time was calculated by the following formula: PDT = (*t*_2_ − *t*_1_)/3.32x (log *n*_2_ − log *n*_1_), where *t* = time in days and *n* = number of cells. In the second approach using MTT, cell lines were seeded in six replicates in 96‐well plates to reach 100% confluence after 7–8 days of culture. The MTT assay was performed every 24 h by adding 0.5 mg·mL^−1^ MTT (M2128; Sigma‐Aldrich) to each well. After 2 h of incubation, the formazan precipitate generated by cells was diluted using a 1 : 3 solution of Glycine buffer (0.1 m NaCl and 0.1 m Glycine) and DMSO to each well. Absorbance was measured at 560 nm in a PowerWave XS microplate spectrophotometer (Biotek, Winooski, VT, USA), and PDTs were calculated using graphpad prism 6 (La Jolla, CA, USA).

##### 2.2.5.2 Percentage of proliferating cells

Cells were seeded in a 12‐well plate in duplicate. When cells reached 50–60% confluence, they were trypsinized and treated according to the Click‐iT^®^ EdU Flow Cytometry Assay Kit (C10425; ThermoFisher Scientific) manufacturer's instructions. A total of 20 000 events were analyzed from each sample using a facs canto II cytometer and modfit LT V.3.3.11 software to obtain the percentage of proliferating cells.

##### 2.2.5.3 Wound healing assay

Cells were seeded in culture inserts (80209; ibidi, Gräfelfing, Germany) to reach confluence after 24 h, and then, culture inserts were removed. Pictures were taken at 0, 4, 8, 12, and 24 h after removal using an optical microscope. Each cell line was seeded in triplicate, and analyses were performed using tscratch software [39]. Proliferation was not inhibited either pharmacologically or with serum deprivation.

##### 2.2.5.4 Colony formation assay

###### 2.2.5.4.1 Two‐dimensional colony formation assay

A total of 300 cells/well were seeded in 12‐well plates in duplicate. After 10 days, cells were fixed with methanol for 10 min and then stained with 0.1% crystal violet for 10 more minutes.

###### 2.2.5.4.2 Three‐dimensional colony formation assay

First, we plated a bottom layer of agar in 6‐well plates, consisting of 1 mL of 1.6% SeaPlaque agar in DMEM, allowing it to solidify at RT for 5 min. Then, we plated the upper layer of 0.8% SeaPlaque agar in DMEM containing 20 000 cells·mL^−1^. Finally, we added 1 mL of DMEM supplemented with 10% FBS and 1% Penicillin/Streptomycin. After culturing for 14 days, cells were fixed and stained with a solution of 0.1% crystal violet in paraformaldehyde for 1 h. The cell lines were seeded in duplicate. Pictures of the colonies were taken using an optical microscope.

#### 2.2.6 *In vivo* tumorigenicity

A total of 1 × 10^7^ cells resuspended in 200 μL of PBS‐Matrigel (ratio 1 : 1) were injected intramuscularly near the sciatic nerve of 6‐week‐old female athymic nude mice. Animals were monitored weekly, and when tumors reached 1 cm in diameter, they were resected. If tumors did not reach this size, they were resected after 3 months. The study received IDIBELL Animal Ethics Experimentation Committee (CEEA‐IDIBELL) (#9111) approval.

### 2.3 Immunohistochemistry marker analyses

Paraffin‐embedded tissues of human primary and passage one PDOX tumor sections (3 μm) were deparaffinized and gradually rehydrated. Endogenous peroxidases were blocked by incubation with hydrogen peroxide (H_2_O_2_ 3% for 20 min), and antigen retrieval was performed by heating tissue sections for 20 min in citrate buffer (pH = 6). Blocking was performed by incubation for 20 min with 10% goat serum. The primary antibodies Vimentin (1 : 200, 180052; Life Technologies, Carlsbad, CA, USA), SOX10 (1 : 50, 383R‐14; Cell Marque, Rocklin, CA, USA), H3K27me3 (1 : 200, 9733S; Cell Signalling, Danvers, MA, USA), CD34 (IR632; DAKO), S100B (1 : 300, Z0311; DAKO), and Ki‐67 (1 : 10, M7240; DAKO) were incubated overnight at 4 °C following the manufacturer's guidelines. The secondary HPRT‐conjugated antibody (EnVision; DAKO) was incubated at RT for 30 min. Finally, staining was conducted using diaminobenzidine (DAB; DAKO) for 10 min; nuclei were counterstained with hematoxylin. Images were taken using a Nikon Eclipse 80i vertical microscope. For immunohistochemistry of cell lines, a cell pellet was generated and incubated with equal volumes of human plasma and thrombospondin (Grifols, Barcelona, Spain), to generate a cell clot that could be embedded in paraffin.

### 2.4 Genomic analyses

Table S2 summarizes the different genomic analyses performed in the patient tumors and the PDOX and cell line models.

#### 2.4.1 DNA and RNA extraction

The GentraPuregene Kit (Qiagen, Hilden, Germany) was used for DNA extraction from frozen human and PDOX tumors, according to the manufacturer's recommendations, after homogenization using TissueLyser II (Qiagen). DNA quality and quantity were assessed by agarose gel, NanoDrop, and Qubit.

For RNA extraction, Direct‐zol RNA MiniPrep (R2050; Zymo Research, Irvine, CA, USA) and TRI reagent (R2050‐1‐50; Zymo Research) were used according to the manufacturer's protocol. RNA quality and quantity (RNA Integrity Number) were assessed by NanoDrop and 4200 TapeStation (Agilent Technologies, Santa Clara, CA, USA).

#### 2.4.2 SNP array

SNP array was performed using BeadChip technology from Illumina (San Diego, CA, USA). All samples (primary tumors, PDOX tumors, and cell lines) were analyzed using HumanOmniExpress‐24v1‐1 (713 599 SNPs), except for those previously described in Castellsagué et al., [32], which were analyzed using Illumina OmniExpress for the SP‐01 primary tumor and Illumina Omni1S for the SP‐01 orthotopic xenograft tumor. Raw data were processed with Illumina Genome Studio to extract B allele frequency (BAF) and log R ratio (LRR) as described previously [32]. We used Genome Alteration Print (GAP) [40] to obtain the copy number (CN) profiles of the samples.

#### 2.4.3 Whole exome sequencing (WES) and whole genome sequencing (WGS)

Whole exome sequencing was performed in primary tumors, the patient's constitutional DNA (except for SP‐06), PDOX tumors at passage one, and cell lines (maximum passage 10). We used the Agilent SureSelect Human All Exon V5 kit (Agilent) according to the manufacturer's instructions. Paired‐end sequencing was performed on a HiSeq2000 instrument (Illumina) using 150‐base reads, and the analysis was performed as described previously [32].

The WGS, only performed in the primary tumors, was produced at BGI (Shenzhen, China). In short, the libraries were prepared following standard DNBseq protocols, sequenced in a BGISEQ‐500 to a median of 881 million 150‐bp paired‐end reads per sample, and mapped with BWA‐MEM [41] against the GRCh38 genome.

#### 2.4.4 Selection of somatic variants using WES and WGS

Whole exome sequencing and WGS data were processed as described in [38]. In summary, small nucleotide variants were called with Strelka2 [42] and annotated with annovar [43]. We ran the somatic calling in those samples (4 individuals) where we had tumor‐normal pair, and the germline calling in all samples followed by filters to enrich in somatic variants. For the somatic variant calling of Strelka2, we followed the developers' recommendations; thus, we first ran the Manta SV and indel caller [44] on the same set of samples, and then, we supplied Manta's candidate indels as input to Strelka2 somatic calling. We used these results to validate the ones obtained by the germline calling.

After running the Strelka2 germline calling in all samples (tumor and normal), we filtered Strelka2 results from WGS data to select potential driver variants affecting protein function by selecting exonic and splicing variants. Then, we filtered out variants with a population frequency (AF_popmax) higher than 1%, classified as benign in ClinVar [45], annotated as benign or likely benign in InterVar automated [46], present in more than 1 individual or classified as pathogenic in less than five out of seven in silico predictors (SIFT pred [47], PolyPhen2 HDIV pred [48], LRT pred, Mutation Taster pred [49], Mutation Assessor pred [49], FATHMM pred [50], and CLNSIG [45]). Then, we filtered out those variants with a variant allele frequency (VAF) lower than 0.1. In addition, we removed nonframeshift deletion or insertion variants present in dbSNP and variants in highly variable genes (*MUC3A*, *MUC5AC*, *OR52E5*, *OR52L1*, *SMPD1*, *PRAMEF*, and *LILR*). Finally, we filtered out the variants present in dbSNP except for those included in the Catalogue of Somatic Mutations in Cancer (COSMIC) (https://ftp.ncbi.nlm.nih.gov/snp/others/rs_COSMIC.vcf.gz) or the International Cancer Genome Consortium (ICGC) (https://ftp.ncbi.nlm.nih.gov/snp/others/snp_icgc.vcf.gz) variant lists. Whole exome sequencing data were processed using the same approach and used to validate the variants identified in WGS data. Moreover, we used Integrative Genomic Viewer (IGV) [51] for performing a manual inspection of TSGs associated with MPNSTs.

#### 2.4.5 Mutational signatures

As previously described in Magallón‐Lorenz et al. [38], raw variants called by Strelka2 in WGS data were used for the mutational signature analysis. Since normal pairs were not available, we applied a series of filters to approximate a somatic callset: we filtered out the variants with a population frequency (AF_popmax) higher than 1%, called in more than one cell line, with a VAF lower than 0.1, and variants in highly variable genes (*MUC3A*, *MUC5AC*, *OR52E5*, *OR52L1*, *SMPD1*, *PRAMEF*, and *LILR*). We also filtered out the variants in dbSNP except for those present in COSMIC and ICGC. We used this call set enriched in somatic variants with the mutSignatures (42) R package to estimate the contribution of each of the 30 COSMIC mutational signatures to the mutational profile of each cell line.

#### 2.4.6 Structural variants detection from WGS

The structural variants (SVs) detection methodology was described previously by Magallón‐Lorenz et al. [38]. LUMPY (41) was used via Smoove (https://github.com/brentp/smoove) as a SV caller with parameters for small cohorts and excluding the problematic regions defined in https://github.com/hall‐lab/speedseq/blob/master/annotations/exclude.cnvnator_100bp.GRCh38.20170403.bed. We also used CliffHunteR (https://github.com/TranslationalBioinformaticsIGTP/CliffHunteR), an in‐house developed sensitivity‐oriented R package for breakpoint detection, and a thorough visual inspection using IGV to detect breakpoints affecting TSGs associated with MPNSTs (*NF1*, *CDKN2A*, *SUZ12*, *EED*, and *TP53*). To discard germline SVs, we filtered out SVs present in the Database of Genomic Variants (DGV) and the SVs with the same breakpoints in more than two tumors.

#### 2.4.7 RNA sequencing

RNA‐seq libraries were established and sequencing of primary tumors was performed at Centro Nacional de Análisis Genómicos (CNAG, Barcelona, Spain), pooling three samples per lane (paired‐end, 2 × 100).

#### 2.4.8 Fusion‐gene detection from RNA‐seq

We applied the default parameters of STAR‐Fusion for the detection of fusion genes from RNA‐seq. After obtaining the results, we performed bibliographic research for looking for potential fusion genes associated with disease.

#### 2.4.9 Methylome profile and Uniform Manifold Approximation and Projection (UMAP) analysis

DNA methylation profiles were generated using the Infinium MethylationEPIC (850 k) BeadChip array (Illumina) according to the manufacturer's instructions. The data were processed as described previously [52]. The two‐dimensional UMAP embedding was created using the 20 000 most variable CpGs from the DNA methylation profiles of the cell lines and the reference cohorts of soft‐tissue tumors 52. The UMAP analysis was performed using the R package umap (version 0.2.7.0) with default parameters except for *n*_neighbors = 8.

### 2.5 *In vitro* drug testing

The half‐maximal inhibitory concentration (IC_50_) of JQ1, MLN8237 (Alisertib), and PD‐0325901 (Mirdametinib; Selleckchem, Houston, TX, USA) was calculated for each cell line, as we described previously [34]. Compounds (stock at 10 mm) were added in three replicates and subsequently diluted fivefold from 100 μm to 0.16 μm. The IC_50_ was calculated using graphpad prism Version 6. For combination assays, the following previously described protocols were performed [34]. Combination Index (CI) values for the combinations were calculated using compusyn software, based on Chou‐Talalay calculations [53]. When CI was < 0.9 at high values of fraction affected of cells (fraction of cell death by treatments), we labeled the combination as synergistic [54].

## 3 Results

### 3.1 Expansion of the MPNST platform: From genuine MPNSTs to other clinically misclassified tumor entities

Six primary tumors (sporadic tumors SP‐01, SP‐04, SP‐05, and SP‐06; and NF1‐derived NF1‐08 and NF1‐09) were identified and removed at the Bellvitge, Vall d'Hebron, and Germans Trias i Pujol hospitals. After surgery, the tumors were sent to the respective pathology services, analyzed following standard methodologies, and classified as MPNSTs, following the WHO classification of soft‐tissue tumors and bone. In parallel, part of each tumor was sent to our laboratory and split for DNA extraction, PDOX engraftment in nude mice, and cell culture expansion (see Section 2).

**Fig. 1:**
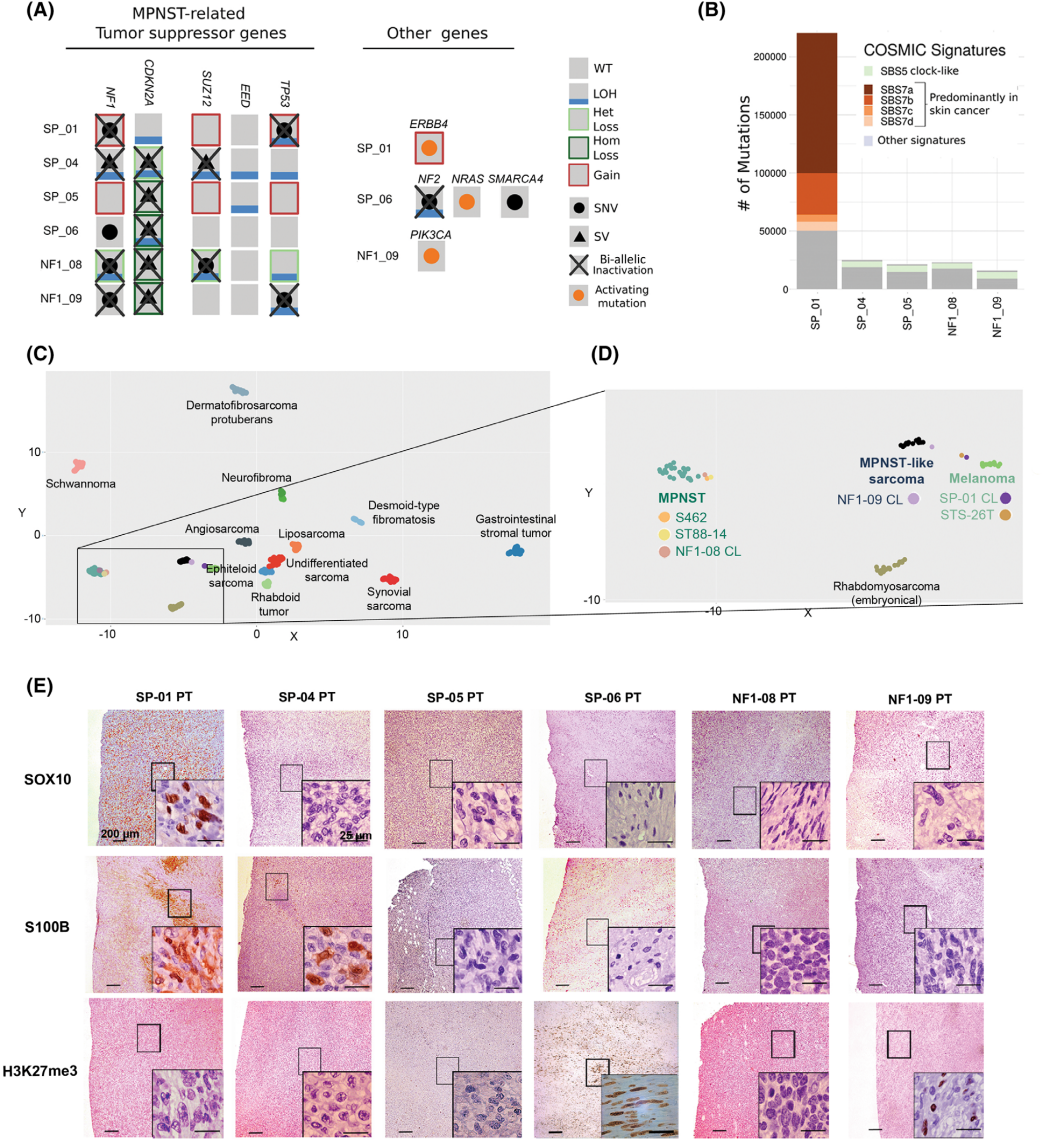
Genomic, epigenomic, and histological characterization of primary tumors and diagnostic validation. (A) Genetic status of the most recurrent inactivated TSGs in MPNSTs using WGS and genes related to other tumor entities. A gray square represents a WT gene; a blue line indicates the presence of LOH; a black dot represents a single nucleotide variant (SNV) affecting the gene; an orange dot represents an activating SNV in the gene; a black triangle indicates a SV; a red square is for CN gain; a light green square is for heterozygous CN loss (Het loss) of the gene, and dark green is for homozygous CN loss (Hom loss); the complete biallelic inactivation of a gene is represented by a black cross. SP‐06 tumor TSGs status was obtained using WES and SNP array. (B) Number of somatic SNVs and contribution of COSMIC mutational signatures in primary MPNSTs. SP‐06 was not included as WGS was not performed on this tumor (*n* = 1). (C) UMAP plot representing methylome classification of multiple sarcomas. Each dot represents a tumor sample and each color a different sarcoma type [52]. (D) Inset amplification of the UMAP plot, showing the classification of the methylome profile of three cell lines derived from our primary tumors (SP‐01, NF1‐08, and NF1‐09) and three other established control cell lines (S462, ST88‐14, and STS‐26T). The MPNST group is represented in blue, the MPNST‐like group in black, and melanomas in green. Each cell line is represented by a unique color. CL: cell line. (E) Representative immunostaining of SOX10, S100B, and H3K27me3 in the patient's primary tumors (*n* = 1). PT: Primary tumor. Original magnifications are 40× and 600× in the inset magnified view, and scale bars are 200 μm and 25 μm, respectively.

To characterize the primary tumors, genomic, epigenomic, and histologic analyses were performed. We used WGS and WES to analyze the status of the most recurrent inactivated TSGs in MPNSTs: *NF1*, *CDKN2A*, and *SUZ12* and *EED* (from PRC2). In addition, we analyzed the status of genes unrelated to MPNSTs (Fig. 1A, Table 1). Only SP‐04 and NF1‐08 have classic MPNST genetic features like *NF1*, *CDKN2A*, and PRC2 inactivated [10, 11, 12]. NF1‐09 presents MPNST genetic features such as the inactivation of *NF1*, *CDKN2A*, and also *TP53*, but with PRC2 active and an activating mutation in the *PIK3CA* gene. The other three sporadic tumor features distanced them further from classic MPNSTs: SP‐01 only has *NF1* mutated, and SP‐05 and SP‐06 have only *CDKN2A* inactivated. Moreover, the SP‐01 tumor presents an activating mutation in the *ERBB4* gene, which is described as a driver of *BRAF* wild‐type (WT) melanomas [55, 56], and SP‐06 presents an oncogenic *NRAS* mutation, inactivation of *NF2*, and a truncating mutation in *SMARCA4* (in one allele; Fig. 1A, Table 1).

**Table 1:** Summary of genetic, epigenetic, and histological marker expression features of the six primary tumors. del, deletion; ger, germline; het, heterozygous; NA, not applicable; som, somatic. Red color shading indicates complete inactivation of the gene. Blue shading indicates positive validation of MPNST diagnosis.

Tumor ID	Other aliases	Tumor (%)	Genetics						Epigenetics	MPNST markers				
			*NF1*	*CDK2NA*	*SUZ12*	*EED*	*TP53*	Number of somatic exonic SNV	Cell line methylome classification	SOX10	S100B	H3K27me3	Additional alterations not related to MPNSTs	Potential tumor classification
SP‐01	MPNST‐SP‐01	50	NM_000267.3:c.3520C > T: p.(Gln1174*) (som)^a^ NM_000267.3:c.3888T > A: p.(Tyr1296*) (som)^a^	WT	WT	WT	NM_000546.6:c.949C > T: p.(Gln317*)^a^ + LOH	1410	Melanoma	+	+	−	***ERBB4***:NM_005235:c.1834C > T: p.(Arg612Trp) (het) **COSMIC signature 7**	Melanoma
SP‐04	MPNST‐SP‐04	89	SV: translocation‐mediated inactivation (som) + LOH	SV: inversion‐mediated del + LOH	NM_015355.2:c.1596‐28_1616del + LOH	WT	WT	20	NA	−	(Focal +) −	−	NA	MPNST
SP‐05	MPNST‐SP‐05	86	WT	SV: inversion‐mediated del	WT	WT	WT	23	NA	−	−	−	***LMNA‐NTRK1*** gene fusion	*NTRK*‐rearranged spindle cell neoplasm
SP‐06^b^	MPNST‐SP‐06	50	WT	SV: del^b^ + LOH	WT	WT	WT	23	NA	−	−	+	***NRAS***:NM_002524.5:c.35G > C: p.(Gly12Ala) (het) ***NF2***:NM_000268:c.1157delA: p.(Lys387Argfs*39) + LOH ***SMARCA4***:NM_003072:c.3121C > T: p.(Gln1041*) (het)	Unclassifiable
NF1‐08	MPNST‐NF1‐08	95	NM_000267.3: c.701_730 + 10del (ger) + LOH	SV: inversion‐mediated del	NM_015355.2:c.1236_1240del: p.(Glu415Glyfs*5)^a^ + LOH	WT	WT	33	MPNST	−	−	−	NA	MPNST
NF1‐09	MPNST‐NF1‐09	62	NM_000267.3:c.6792C > A: p.(Tyr2264*) (ger)^a^ NM_000267.2: c.1186‐5_1186‐1del (som)	SV: inversion‐mediated del	WT	WT	NM_000546.6:c.844C > T: p.(Arg282Trp)^a^ + LOH	NA	MPNST‐like	−	−	+	***PIK3CA***:NM_006218.4:c.1035T > A: p.(Asn345Lys) (het)	MPNST

^a^ The SNV is present in the primary tumor, PDOX tumor, and cell line (in the case of SP‐01, NF1‐08, and NF1‐09).

^b^ SP‐06 tumor data obtained from WES and SNP array.

Bold terms are highlight the genetic alterations not related to MPNSTs in the six analyzed primary tumors.

The mutational frequency and signatures of all tumors except SP‐06 (in which we only performed WES) were analyzed using WGS. We observed that tumor SP‐01 exhibited at least a ninefold higher mutation number compared with other primary tumors, mainly containing the SBS7 COSMIC mutational signature, characteristic of skin cancers [57]. The other tumors presented low mutation burden and no specific COSMIC signatures besides clock‐like signature 5, which appears in most tumor types [57] (Fig. 1B).

Moreover, the methylome profile of the three cell lines obtained from tumors SP‐01, NF1‐08, and NF1‐09 was compared with other sarcomas. Figure 1C presents a methylome profile classifier of several sarcomas using a UMAP plot [52]. Taking a closer view of the MPNST region, SP‐01's methylome profile matched that of melanoma (like STS‐26T, which was recently reclassified from an MPNST to a melanoma cell line [38]), tumor NF1‐08 clustered with the classic MPNST group (as for ST88‐14 and S462 cell lines), and NF1‐09 clustered in the rather catchall MPNST‐like sarcoma group (Fig. 1D, Table 1). This group of sarcomas is characterized by bearing an active PRC2, which generates a different methylation pattern compared with PRC2‐inactivated tumors [58].

Finally, we analyzed several markers routinely clinically used for MPNST diagnosis: S100B and SOX10 (cell identity markers of the peripheral nervous system), H3K27me3 (epigenetic marker of PRC2 dysfunction), Vimentin (mesenchymal cell marker), Ki‐67 (proliferation cell marker), and CD34 (fibroblast and endothelial marker). Only tumor SP‐01 presented strong dual staining for S100B and SOX10, contrary to classic MPNSTs that present negative or focal expression [59], like S100B expression in the SP‐04 tumor. We found a lack of H3K27me3 in four tumors, including SP‐01 and SP‐05 (Fig. 1E, Table 1), which are WT for *SUZ12* or *EED* (Fig. 1A), implying that PRC2 inactivation may be due to other genetic alterations unrelated to MPNSTs [12]. All samples were positive for the soft‐tissue tumor marker Vimentin, as expected, and the CD34 endothelial cell marker was negative in tumor cells, only marking vessels (Fig. S1).

Taken together, only SP‐04 and NF1‐08 fulfilled most genetic features of classic MPNSTs and evidence shows that three primary tumors may be misdiagnosed as MPNSTs (Table 1). Tumor suppressor gene profile inactivation, mutational burden and signatures, methylome profile classification, and positive expression of neural crest markers may indicate that SP‐01 should be reclassified as a melanoma. In the case of tumors SP‐05 and SP‐06, besides the TSG inactivation pattern, they presented specific genetic features that do not correlate with MPNSTs. SP‐05 tumor bore the fusion‐gene *LMNA‐NTRK1*, which was identified using WGS and confirmed by RNA sequencing. The fusion product was histologically validated by overexpression of *NTRK* as the tumor retained the kinase domain of *NTRK1* in exons 13 to 17 (Fig. S2). Currently, there are no recurrent fusion genes described in MPNSTs [60, 61], potentially indicating that tumor SP‐05 may be an *NTRK‐*associated sarcoma. Finally, tumor SP‐06 presented genetic alterations in genes *NF2*, *SMARCA4*, and *NRAS*, which may point to other tumor entities.

With all the genomic data generated, an independent pathologist analyzed the hematoxylin/eosin staining of the primary tumors not validated as classic MPNSTs (Fig. S3), confirming NF1‐09 as a high‐grade MPNST, SP‐01 as a melanoma, SP‐05 as an *NTRK*‐associated spindle cell sarcoma, and SP‐06 remained unclassifiable.

### 3.2 Expansion of the MPNST platform: Generation of PDOX models and new cell line models

**Fig. 2:**
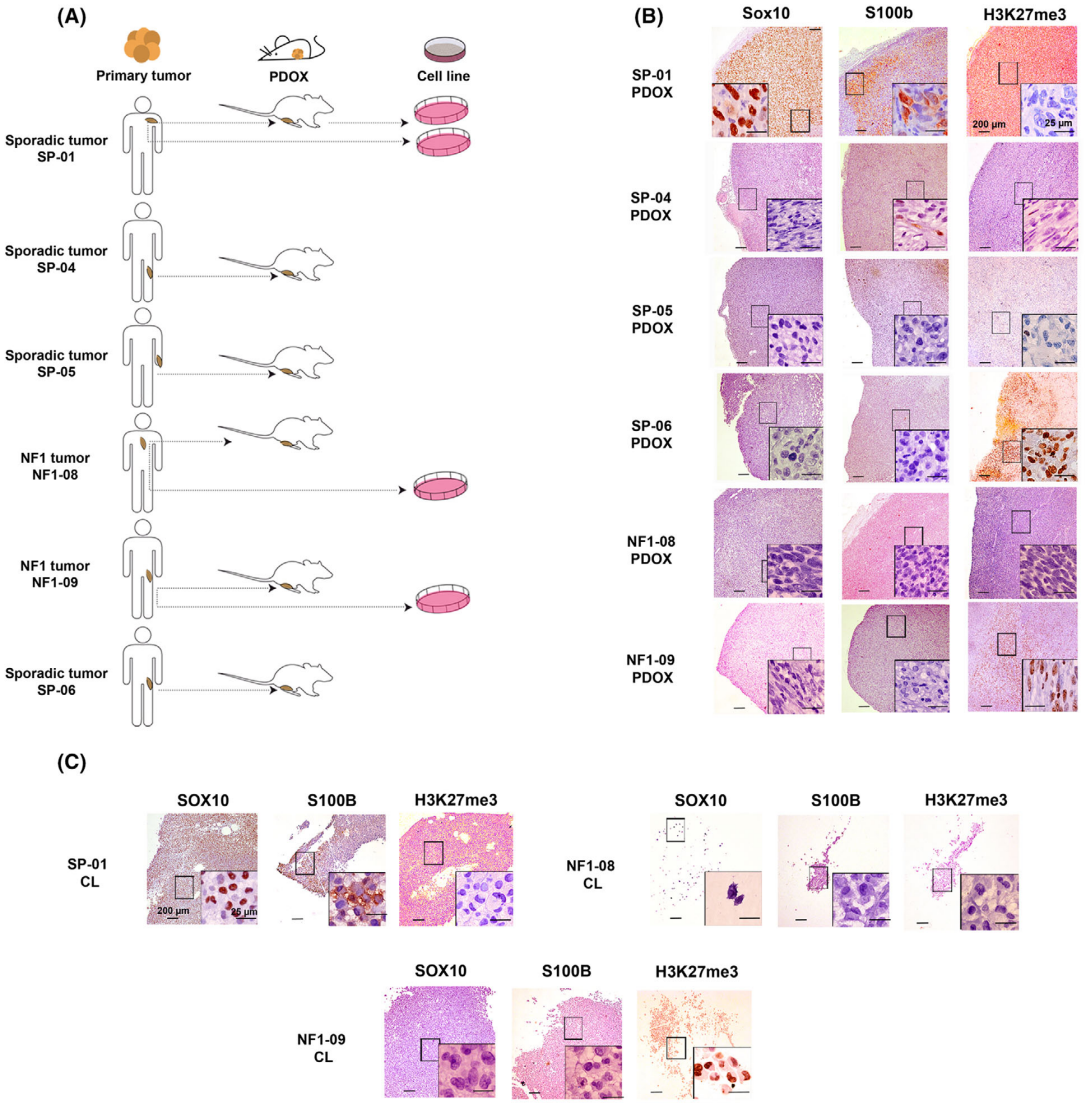
Characterization of new PDOX and cell line models. (A) Scheme of the *in vitro/in vivo* models generated from the patient's tumors. Two cell lines were generated from the same patient, one from the primary tumor and the second from the PDOX tumor. (B) Representative histological stains of Sox10, S100b, and H3K27me3 in the six PDOX tumors (*n* = 1). Original magnifications are 40× and 600× in the inset magnified view, and scale bars are 200 μm and 25 μm, respectively. (C) Representative histological stains of SOX10, S100B, and H3K27me3 in the three cell lines derived from primary tumors (*n* = 1). CL: cell line. Magnifications are 40× and 600× in the inset magnified view, and scale bars are 200 μm and 25 μm, respectively.

From the six primary tumors, we were able to obtain PDOX models for each engrafted tumor and, in addition, three new cell lines, two from NF1‐MPNST tumors (NF1‐08 and NF1‐09), and one from the sporadic tumor SP‐01, a suspected melanoma. Moreover, a fourth cell line was generated from the SP‐01 PDOX model (SP‐01‐OT; Fig. 2A). Remarkably, we obtained a total of three pairs of *in vitro/in vivo* models for primary tumors, only two of which were true MPNSTs, from the same patient. DNA microsatellite authentication analysis demonstrated that the newly generated cell lines and PDOX models matched blood and primary tumor profiles from patients (Table S3).

The mouse PDOX models presented the main histological features of the primary tumors, such as spindle cell hypercellularity with fusiform nuclei (Fig. S3). We performed a thorough histological characterization of PDOX models and cell lines, testing the same MPNST histological markers as in the primary tumors. We found a high correlation between primary tumors, PDOX tumors, and cell lines in terms of marker expression (Fig. 2B,C, Table 2, Fig. S1). The only differences were observed for Ki‐67 staining: The tumor cell proliferation rate was similar independent of whether the tumor was primary or orthotopic (ranging from 10 to 30% of proliferating cells); however, it was slightly increased in cell lines (40 to 60%), probably due to the intrinsic nature of cell cultures (Fig. S1, Table 2).

**Table 2:** Summary table comparing histological marker expression in primary tumors (PTs), PDOX tumors (OTs), and cell lines (CL).

Marker	SP‐01			SP‐04		SP‐05		SP‐06		NF1‐08			NF1‐09		
	PT	OT	CL	PT	OT	PT	OT	PT	OT	PT	OT	CL	PT	OT	CL
SOX10	+	+	+	−	−	−	−	−	−	−	−	−	−	−	−
S100	+	+	+	− (Focal)	− (Focal)	−	−	−	−	−	−	−	−	−	−
H3K27me3	−	−	−	−	−	−	−	+	+	−	−	−	+	+	+
CD34	−	−	−	−	−	−	−	−	−	−	−	−	−	−	−
Vimentin	+	+	+	+	+	+	+	+	+	+	+	+	+	+	+
Ki‐67 (%)	35	30	60	10	40	20	30	5	10	30	30	60	10	10	40

Finally, in the process of obtaining tumor cell lines from tumors SP‐04, SP‐05, and SP‐06, we observed only tumor‐associated fibroblast isolation, evidenced by SMA‐positive cells with diploid DNA content and no structural abnormalities in the genome (Fig. S4). These cell lines were also included in the platform for further characterization in the future.

### 3.3 New cell lines and PDOX models recapitulate the main genomic features of primary tumors

A thorough genomic characterization of PDOX models (at passage one) and cell lines was performed using SNP array and WES (Table S2), for validation against primary tumors.

**Fig. 3:**
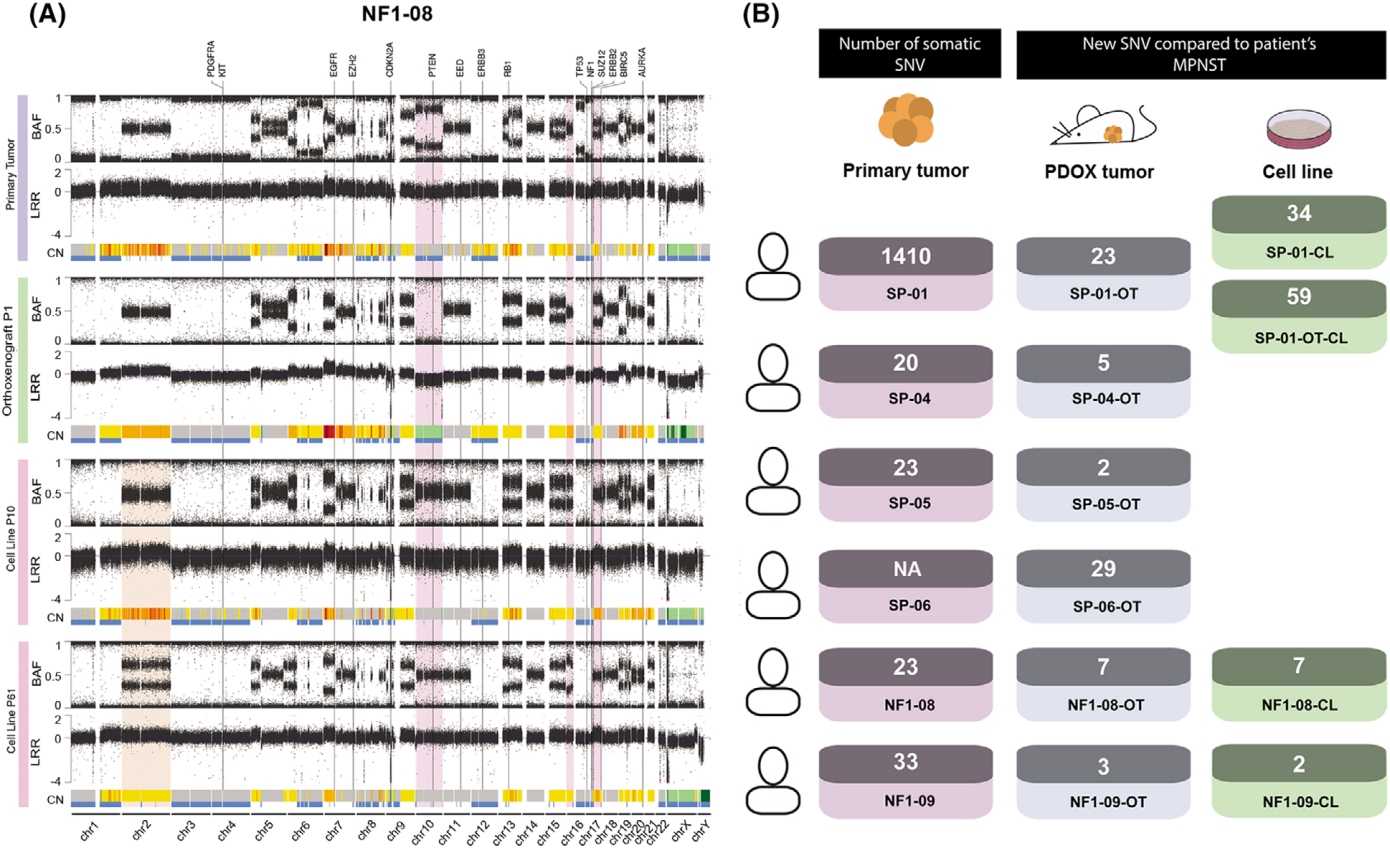
Patient‐derived orthotopic xenografts and cell lines recapitulate the main genetic and genomic features of primary tumors. (A) CN profile of primary tumor, orthoxenograft (PDOX) tumor, and cell line from patient NF1‐08, representing that the models recapitulate the genomic hallmarks of the primary tumor. BAF and LRR profiles are represented. CN variations are represented by a colored line under each LRR: gray for 2n region; yellow to red for >2n, representing chromosomal gain; and green for <2n, representing chromosomal loss. LOH events are shown in blue. Genomic differences between primary and xenograft tumors are highlighted in purple, and differences between cell lines at low and high passage are marked in a cream color. (B) Number of somatic SNVs in the coding regions of primary tumors and models calculated using WES. The blood of patients was used as a control of constitutive DNA. New somatic SNVs were calculated in PDOX tumors and cell lines compared with primary tumors. The number of SNVs of primary tumor SP‐06 was not analyzed due to the lack of blood sample from the patient. OT: PDOX tumor.

A hallmark characteristic of MPNSTs is the presence of hyperploid and highly altered genomes [7]. Using SNP arrays, we analyzed the CN profile and allele ratios of primary tumors, PDOXs, and cell lines. The data proved that the genomic structure of tumors SP‐04 and NF1‐08 highly resembled that of classic MPNSTs, presenting gains of whole chromosomes or large chromosomal regions and a few losses of genetic material, alongside extended regions of loss of heterozygosity (LOH; Fig. 3A, Fig. S5) [38]. Tumors SP‐01 and SP‐05, potentially reclassified as other tumor entities, nonetheless, also presented similar classic MPNST genomic features. Contrarily, tumors NF1‐09 and SP‐06 presented less‐altered genomes (Fig. S5).

Genome features from PDOX tumors and cell lines highly recapitulated the patient tumors, thus validating our models (Fig. 3A, Fig. S5). There were only a few differences, probably due to loss of signal from human stromal cells in the PDOX or cell lines. We found more differences between cell lines and primary tumors than PDOX and primary tumors, concordant with the intrinsic characteristics of cell culture conditions. Moreover, we checked the genome stability of cell lines through the passages. We found a high degree of genomic stability in all cases, except for cell line NF1‐09 (Fig. 3A, Fig. S5). This cell line exhibited a different genomic structure profile in late passages compared with early passages, the latter being more similar to primary and PDOX tumors. The changes observed in the passages could be explained by the selection of a specific subpopulation that seemed to exist in the primary tumor but in a small proportion. Remarkably, the cell line genome at high passage number bears a greater resemblance to the genomic features of MPNST [60] (Fig. S5).

Using WES, we were able to confirm the presence of all somatic single nucleotide variants (SNVs) identified in primary tumors, in their corresponding PDOX and derived cell lines (Table 1). We also quantified somatic SNVs and small indels in coding regions of primary tumors and matched PDOX models and cell lines, to analyze the genetic variation caused by new somatic variants in models compared with primary tumors. We performed a somatic calling of all samples, except for the SP‐06 tumor as we did not have a normal counterpart. Excluding SP‐01 (due to its higher mutation burden compared with other tumors), PDOX tumors at first passage, just after engraftment, presented a mean of ~ 9.2 (2–29) new somatic variants compared with primary tumors (Fig. 3B); cell lines, at low passage, presented a mean of ~ 4.5 (2–7) new variants. The SP‐01 PDOX tumor only presented 23 new SNVs compared with the primary tumor, similar to the two cell lines derived from this tumor that showed a mean of ~ 46.5 (34–59) new SNVs (Fig. 3B). Altogether, the low number of new somatic variants detected in the engrafted tumors and cell lines with respect to their primary counterparts reinforces our observation that the models generated in this study faithfully recapitulate the genomic characteristics of the primary tumors, being quite stable genetically.

### 3.4 Tumor‐derived cell lines exhibit heterogeneity in phenotypic and functional features

**Fig. 4:**
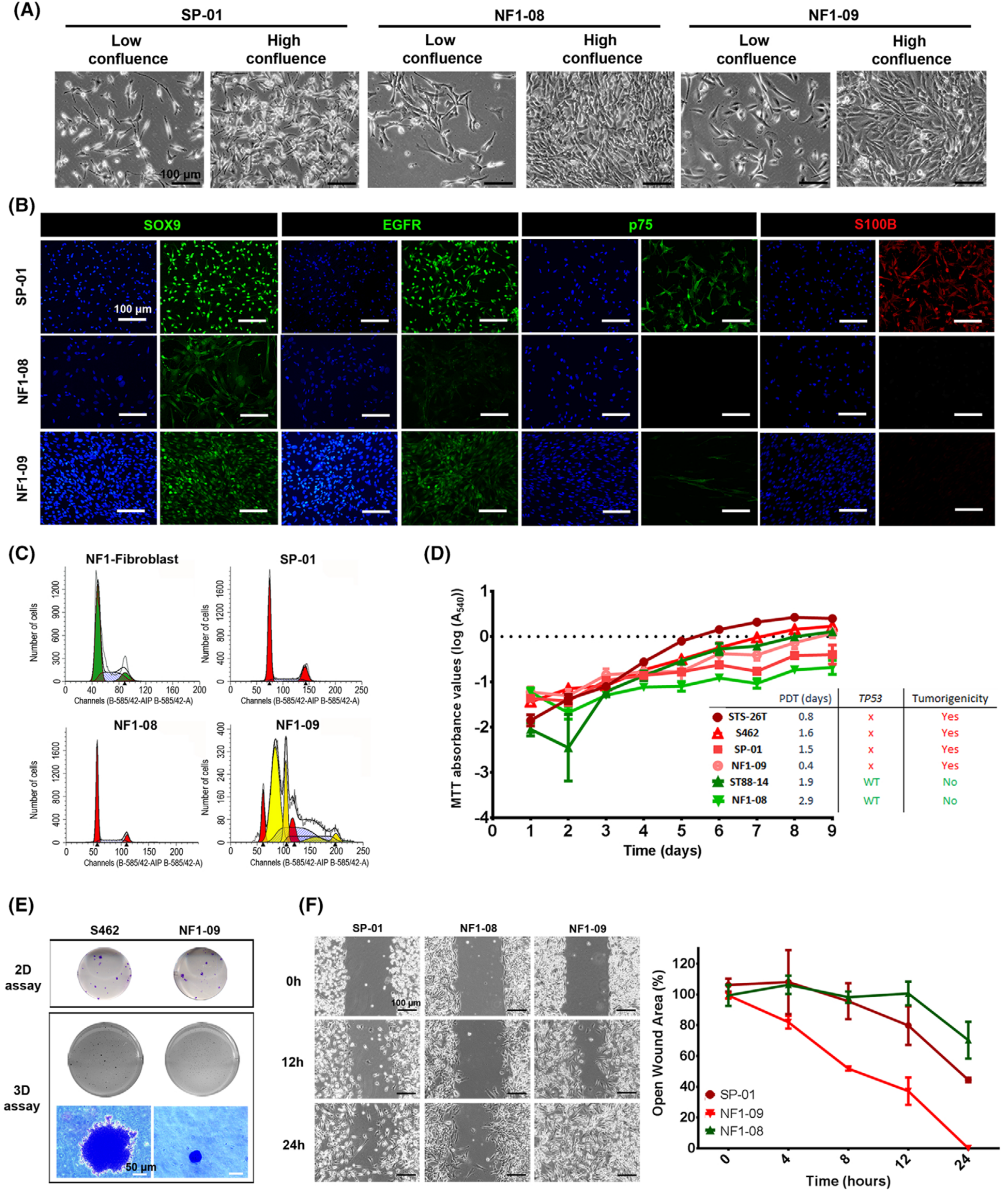
Phenotypic and functional characterization of new established cell lines. (A) Representative morphology images of the newly generated cell lines at low and high confluence (*n* = 1). Images were taken by optical microscope at 100× magnification. The scale bar is 100 μm. (B) Representative immunofluorescence images of MPNSTs markers SOX9 and EGFR, and neural crest‐Schwann cell lineage markers p75 and S100B (*n* = 1). Original magnification of images is 200×, and the scale bar is 100 μm. (C) DNA content analyses of the three cell lines (using fibroblasts derived from a NF1 patient as a diploid control), represented as the number of cells versus DNA quantity (*n* = 2). (D) Cell growth curves of the three newly generated cell lines and three control tumor cell lines (S462, ST88‐14, and STS‐26T), obtained using MTT viability assay. Growth curves are derived from mean values ± SD (error bars, *n* = 6). PDT values and *TP53* status correlate with the tumorigenic capacity of the cell lines. In red, cell lines that generate tumors in mice, with low PDT values and *TP53* inactivated. In green, cell lines that do not generate tumors, with high PDT values and active *TP53*. (E) Colony formation ability of cell lines. Representative images of 2D and 3D colonies generated by the new established cell line NF1‐09 and control MPNST cell line S462; both generate tumors in mice (*n* = 2). Original magnification of images is 400×. The scale bar is 50 μm. (F) Wound healing assay of the three cell lines. Representative images of wound closing were captured at 0, 12, and 24 h (left) at 100× magnification. The migration ability of cells was represented as the percentage of open wound at 0, 4, 8, 12, and 24 h (right). Open wound curves are derived from mean values ± SD (error bars, *n* = 3). In red, cell lines that generate tumors in mice and, in green, cell lines that do not generate tumors. The scale bar is 100 μm.

We performed a comprehensive characterization of the three cell lines (SP‐01, NF1‐08, and NF1‐09) isolated from primary tumors. The first set of analyses aimed to describe the cells' phenotypic characteristics in the different cell cultures, as regards morphology (Fig. 4A), marker expression (Fig. 4B), and ploidy (Fig. 4C). Morphologically, the two cell lines from NF1‐MPNST validated tumors (NF1‐08 and NF1‐09) were composed of small, polygonal cells that grew forming a monolayer without contact inhibition, similar to the morphology of other classic MPNST cell lines like S462 or ST88‐14 (Fig. S6A). Regarding the expression of neural crest stem cell lineage markers (S100B and p75) and MPNST markers (SOX9 and EGFR) [62, 63], these two cell lines were positive for SOX9 and EGFR expression and negative for S100B and p75 (only focal in the NF1‐09 cell line), similar to MPNST control cell lines (S462 and ST88‐14) [38] (Fig. 4B, Table 3, Fig. S6B). In the case of SP‐01, cells presented Schwann cell characteristics such as bipolar or tripolar morphology with oval nuclei [64] and were positive for all four markers (Fig. 4A,B, Table 3), compatible with a melanoma cell line [65, 66, 67, 68]. As for DNA content, different degrees of aneuploidy were observed across tumor cell lines. The NF1‐08 cell line was between 2n and 3n, while SP‐01 was triploid. In the case of NF1‐09, cell cycle analysis proved that this cell line presented different subpopulations of tumor cells, with heterogeneity of ploidies (Fig. 4C, Table 3). The analyses of several isolated clones indicated the presence of three different cell subpopulations: one higher than 2n; a second nearly triploid; and a third completely tetraploid. The three subpopulations remained stable across multiple passages in culture (Fig. S6C).

**Table 3:** Summary table comparing phenotypic and functional features of MPNST expression in newly generated cell lines and established control cell lines. OT, PDOX tumor; PT, primary tumor; WT, wild‐type.

	Expression of markers				Proliferation		Colony formation		Migration	Genetics		Tumorigenicity
	S100	p75	SOX9	EGFR	PDT (days) MTT	% Proliferating cells	2D‐colony assay	3D‐colony assay	% of open wound (12 h)	*TP53* status	DNA content	
Newly generated cell lines												
SP‐01	+++	++	+++	+++	1.53	20.30	−	−	75.19	Inactivated	>2n	+
SP‐01‐OT	+++	+	+++	+++	2.72	17.75	−	−	55.52	Inactivated	>2n	−
NF1‐08	−	−	++	+	2.90	9.85	−	−	100	WT	>2n	−
NF1‐09	−	− (+ focal expression)	+++	+++	0.44	36.55	+	+	37.44	Inactivated	>2n, 3n and 4n (3 subpopulations)	+
Established cell lines												
STS‐26T	−	−	+++	+++	0.80	53.95	+	+	19.72	Inactivated	4n	+
S462	−	−	+++	+++	1.61	42.40	+	+	79.46	Inactivated	>2n	+
88‐14	−	−	+++	+++	1.59	35.75	+	−	17.59	WT	>2n	−

We further characterized the three cell lines by performing a set of functional assays (summarized in Table 3): proliferation assays (calculating the PDT); *in vivo* tumor formation capacity (Fig. 4D); 2D and 3D *in vitro* colony formation capacity (Fig. 4E); and migration ability (wound healing assay; Fig. 4F). We compared the functional properties of the newly isolated cell lines with those already established (S462, ST88‐14, and STS‐26T).

We calculated the PDT using two different methodologies, Trypan Blue dye exclusion and MTT viability assay, obtaining similar results (Fig. 4D, Fig. S6D). Cell lines STS‐26T, S462, SP‐01, and NF1‐09 had the highest proliferation rates with PDT values < 1, correlating with cell lines that generated tumors in athymic nude mice. Interestingly, these four cell lines all had the *TP53* gene inactivated (Fig. 4D, Table 3). Quantification of proliferating cells in cell lines ranged from 20 to 50%, where lower PDT represents higher rates of dividing cells, as expected (Table 3). Regarding colony formation capacity, only cell lines NF1‐09, S462, and STS‐26T formed colonies in 2D and 3D assays, also generating tumors when engrafted in mice (Fig. 4E, Fig. S6E, Table 3). Cell line SP‐01 was also able to generate tumors *in vivo* (Table 3); however, it was not able to generate colonies in 2D and 3D (not shown). The migration ability also differed between cell lines, SP‐01 and NF1‐09 presenting a higher migration rate in the wound healing assay, similar to STS‐26T and S462 (Fig. 4F, Fig. S6F, Table 3). Contrarily, NF1‐08 had a much lower proliferation capacity, similar to ST88‐14 (PDT values of 2–3 days) and the migration capacity was low, having 100% of open wound area at 12 h. Interestingly, both NF1‐08 and ST88‐14 cell lines did not generate 3D colonies. Moreover, these two cell lines, that were unable to form tumors *in vivo* after engraftment, bore a WT *TP53* (Table 3).

Remarkably, we obtained two cell line models from the same patient (SP‐01), one from the primary tumor and the other from the PDOX (SP‐01‐OT). We characterized and compared the two cell lines (Fig. S7, Table 3), which presented similar features, indicating the utility of isolating cell lines from PDOX.

### 3.5 Different *in vitro* therapeutic responses between genuine MPNSTs and reclassified entities

Finally, the three newly generated cell lines derived from primary tumors and the S462 cell line (as a classic MPNST cell line control) were treated with three different compounds. Two of these targeted pathways deregulated due to the loss of specific TSGs in MPNSTs: the MEK inhibitor Mirdametinib (PD0325901), to compensate the activation of the Ras/MAPK pathway by *NF1* inactivation [14], and the bromodomain inhibitor JQ1 for PRC2 inactivation [13]. The third compound tested was the Aurora A kinase inhibitor (AURKAi) Alisertib (MLN8237), which was previously described to be a good treatment candidate for MPNSTs [69, 70].

**Fig. 5:**
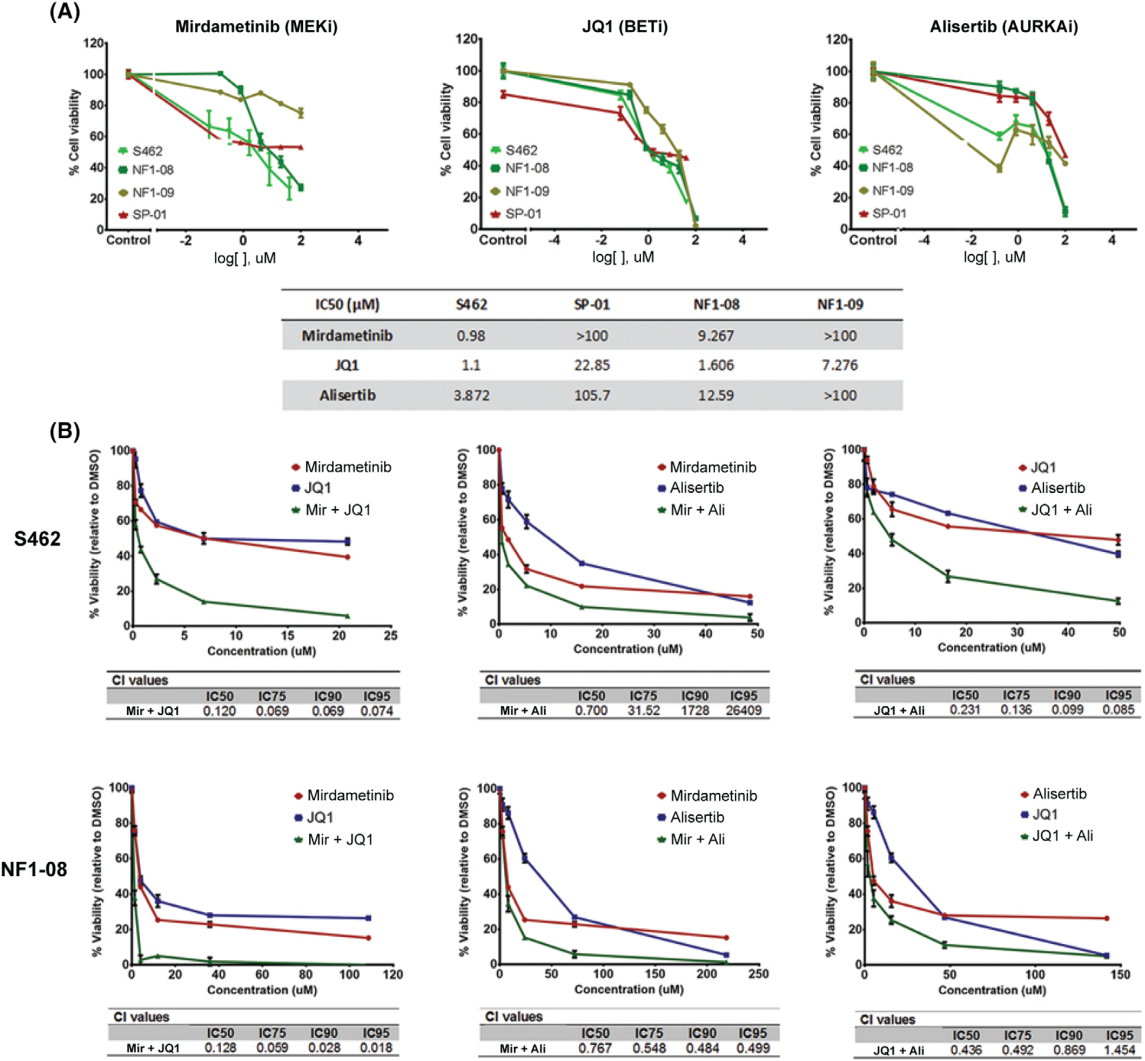
Genuine and confounded MPNST cell lines exhibit different treatment responses. (A) Cell viability plots of cell lines treated singly with MEK inhibitor (MEKi) Mirdametinib, Aurora A kinase inhibitor (AURKAi) Alisertib, and BET inhibitor (BETi) JQ1, and IC50 values of the compounds for each cell line. Cell viability curves are derived from mean values ± SD (error bars, *n* = 3). In green, cell lines S462 and NF1‐08 are classic MPNSTs; in khaki green, the NF1‐09 cell line is an MPNST with active PRC2; and in red, the SP‐01 cell line is derived from a melanoma. (B) Cell viability plots of NF1‐08 and S462 cell lines treated with pairwise combinations of the three compounds, alongside CI values to evaluate the synergy of the combinations. Cell viability curves are derived from mean values ± SD (error bars, *n* = 3). Synergy is observed at CIs <1. Single treatments are marked in blue and red, and combination in green in the cell viability plot.

Classic MPNST cell lines (S462 and NF1‐08) carrying the three recurrent inactivated TSGs (*NF1*, *CDKN2A*, and PRC2) were the most sensitive to the three compounds, which highly decreased cell viability. Surprisingly, for NF1‐09, an MPNST that presents active PRC2, we observed that JQ1 was effective. Furthermore, although *NF1* is completely inactivated in this cell line, we observed a limited therapeutic response to MEKi, probably due to a potential bypass produced by the oncogenic *PIK3CA* mutation present in this cell line. Finally, the suspected melanoma cell line SP‐01 presented a lower response to the three treatments, only reducing cell viability by half at the maximum concentrations of the compounds tested (Fig. 5A). Indeed, NF1‐09 (nonclassic MPNST) and SP‐01 (melanoma) presented higher IC_50_ values for Mirdametinib and Alisertib (> 100 μm) compared with NF1‐08 (9.267 and 12.59, respectively) and S462 (0.98 and 3.872; Fig. 5A). We then wanted to test whether the compounds could be synergistic in combination in the S462 and NF1‐08 cell lines, the only ones sensitive to the single treatments. Both cell lines presented synergistic effects with the three tested combinations, especially for JQ1 plus Mirdametinib, as observed by CI values <1 in a high fraction of affected cells (Fig. 5B). Taken together, we observed that treatment response was different when we used compounds directed against altered MPNST pathways in classic MPNST cell lines compared with other potential tumor entities, such as melanomas.

## 4 Discussion

More than 50% of MPNSTs arise in NF1 patients, being the main cause of early mortality in young patients with this genetic condition [6]. The low prevalence of MPNSTs in the general population hampers the development of therapeutic approaches designed *ad hoc* for this tumor type, making the use of *in vitro* and *in vivo* models paramount to moving toward precision and personalized therapeutic strategies. Malignant peripheral nerve sheath tumors may be difficult to diagnose as other tumor entities can mimic their morphology and marker expression patterns, especially outside the NF1 clinical context [22].

It was recently described that some cell lines commonly used by the scientific community as MPNST cell models, particularly sporadic models, may not be derived from MPNSTs but rather from other entities. This work has drawn attention to the potential heterogeneity of tumors placed under the MPNST umbrella, highlighting the potential utility of genomic and epigenetic information in guiding their differential diagnostics [38]. Thus, we aimed to enrich our precision medicine platform by reclassifying tumor entities that may be confounded using the current clinical tools to diagnose MPNSTs and may be more appropriately classified using genomic, epigenomic, and marker expression information. After the analyses of six primary tumors, we have classified two tumors as classic MPNSTs (the sporadic SP‐04 and the NF1‐related NF1‐08) since both bore the complete inactivation of *NF1*, *CDKN2A*, and *SUZ12* [10, 11, 12, 71, 72] and displayed an MPNST‐compatible genomic CN profile [10, 11]. A third tumor, NF1‐09, was classified as an MPNST although it has PRC2 active, which might account for the NF1‐09‐derived cell line clustering in the MPNST‐like sarcoma group when using a methylome classifier. Moreover, these three MPNSTs presented few somatic SNVs (20–30), similar to other groups described in this tumor type (median of 40–60 variants) [10, 11] and presented negative SOX10 and S100B staining, as expected [24, 73, 74, 75]. A second analysis by an independent pathologist identified tumor NF1‐09 as a high‐grade MPNST.

Remarkably, the other three primary tumors, all sporadic (representing three out of four sporadic cases), after compiling genomic information and re‐evaluation by an independent pathologist, were reclassified as a melanoma (SP‐01), an *NTRK*‐related spindle cell neoplasm (SP‐05), and the third was discarded as an MPNST although further classification was inconclusive (SP‐06). SP‐01 highly mimicked MPNSTs histologically but expressed S100B, p75, and SOX10 neural crest markers, like melanomas [65, 76], and presented a high mutation frequency, mostly associated with the skin cancer COSMIC mutational signature [77]. SP‐05 presented genomic and histological features compatible with MPNSTs but bore an *NTRK*‐associated gene fusion (*NTRK1‐LMNA*). Gene fusions involving the *NTRK* gene family (*NTRK1*, *NTRK2*, and *NTRK3*) are usually described in a broad spectrum of mesenchymal tumors [78]. For instance, *LMNA‐NTRK1* has been reported in Lipofibromatosis‐Like Neural Tumors, which highly resemble low‐grade MPNSTs [79, 80]. A case report study detected this gene fusion within a subset of NF1‐related MPNSTs [81]; however, the histological and molecular characterization of these tumors was scarce. Finally, according to genomic characteristics, tumor SP‐06 is clearly distinct from classic MPNSTs (*NF2* inactivation, *NRAS* oncogenic mutation, and truncating mutation in *SMARCA4*). However, a second analysis by an independent pathologist was unable to provide a definitive identity, highlighting the difficulty in diagnosing MPNSTs and related tumors with overlapping histological characteristics. Other high‐grade sarcomas can also mimic histological and marker expression patterns of MPNSTs, such as synovial sarcoma, fibrosarcomatous dermatofibrosarcoma protuberans, myxofibrosarcoma, or spindle cell sarcomas [82]. Thus, two messages can arise from our work. First, the thorough genomic and histologic characterization of tumors applied in a larger number of samples may facilitate a correct diagnostic of tumors currently labeled as MPNSTs in the clinics. Second, we should reinterpret results obtained with newly rediagnosed models previously considered MPNSTs, such as the STS‐26T cell line (recently reclassified as probably being a melanoma [38]) and used by many different laboratories; or the SP‐01 cell line (also MPNST‐SP‐01 in previous works) [32, 34], used in our group.

Our platform also includes cellular and mouse models from MPNSTs and confounded tumor entities. We have generated PDOXs from all six primary tumors and cell lines from half of them (SP‐01, NF1‐08, and NF1‐09). One of the main challenges was the generation of tumor‐derived cell lines, which is normally difficult to achieve [83] and was not feasible for all primary tumors. Histological and genomic analyses validated that all derived PDOXs and cell lines genuinely represented their respective primary tumors. In the context of the primary tumors studied herein, PDOX generation seems more efficient than establishing 2D cell lines. Interestingly, after 2D establishment, only cell lines with *TP53* inactivated, among other features, had tumorigenic capacity in animal models, although further experiments will be required to elucidate any causal relationship. Altogether, there seem to be different molecular requirements for *in vitro* or *in vivo* model generation. The migration and growth capacities of the cells are also described as hallmarks of the tumor's potential invasion and metastatic capacity [84], being factors that may improve the ability of cells to generate tumors in mice. Beyond *TP53* status, we found a good correlation between *in vitro* and *in vivo* properties. Cell lines with high proliferation, invasion, and migration potential (NF1‐09, SP‐01, and the established cell lines S462 and STS‐26 T), were those exhibiting tumor formation capacity.

Finally, we investigated potential differences in drug treatment response in our three isolated cell lines, which are representative of the clinical diversity of MPNSTs, with classic MPNST cell lines, an MPNST cell line with active PRC2, and a cell line from a tumor entity (melanoma) potentially misclassified as an MPNST. The cell lines were used for testing three targeted drug compounds for MPNSTs: a MEK inhibitor (Mirdametinib or PD0325901), a bromodomain inhibitor (JQ1), and an Aurora A kinase inhibitor (Alisertib or MLN8237). The sensitivity of the cell lines to the compounds was quite different, as expected, as we know that part of these differences arise because we are testing different entities with distinct genetic alterations. Only the classic MPNST cell line NF1‐08 and the S462 cell line were sensitive to the three treatments. NF1‐09, considered nonclassic MPNST, had differences in treatment response compared with the classic ones as it was resistant to AURKAi and MEKi treatments, although the latter could be related to the presence of an oncogenic mutation in *PIK3CA*, and it was sensitive to JQ1, despite being PRC2 WT. The cell line SP‐01 was the most resistant to the three MPNST‐directed treatments, as expected considering it is probably a melanoma cell line. In summary, part of the different responses to single treatments could be attributed to the genetic status of the cell lines; however, this is clearly not the only factor playing a role in drug response. For all combinations, co‐treatment therapies in NF1‐08 and S462 MPNST cell lines generated a synergistic effect, reinforcing this strategy [85].

## 5 Conclusions

In summary, here we present our MPNST precision medicine platform, an excellent tool for research and preclinical studies to reclassify clinically diagnosed MPNSTs. It is noteworthy that we have *in vitro* and *in vivo* pairs from the same primary tumor for two of the three true MPNSTs in this study. Moreover, the expansion of the MPNST platform to tumor entities that might be confounded in routine clinical diagnostics makes it more representative of a real clinical scenario and will constitute a useful tool to obtain correct preclinical information to guide successful clinical trials in humans. The clinical diversity of tumors, together with their specific genetic and genomic alterations, was translated into different response to treatments.

## Conflict of interest

The authors declare no conflict of interest.

## Author contributions

EC‐B, JF‐R, ES, and CL were involved in study concept and design and original manuscript draft. EC‐B, JF‐R, MM‐L, SO‐B, SN‐R, MP, MM, HM, MC, and AV were involved in experimental work acquisition, analysis, and interpretation. MM‐L and BG were involved in bioinformatic analyses. TL and DR were involved in methylome analysis and sarcoma classifier. CR, AE, DPS were involved in clinical case contribution. CR, TMS, and MP were involved in pathology review. All authors were involved in review and editing of manuscript.

### Peer review

The peer review history for this article is available at https://www.webofscience.com/api/gateway/wos/peer‐review/10.1002/1878‐0261.13534.

## Supporting information


**Fig. S1.** Histological stains of Ki‐67, CD34, and Vimentin in the primary tumors and models.
**Fig. S2.** *LMNA‐NTRK1* gene fusion in the SP‐05 tumor.
**Fig. S3.** Hematoxylin–Eosin (H&E) stains of primary tumors and tumors from PDOX mice.
**Fig. S4.** Tumor‐associated fibroblasts derived from three primary tumors.
**Fig. S5.** Copy number profile of primary tumor, orthoxenograft (PDOX) tumor, and cell line from tumors SP‐01, SP‐04, SP‐05, SP‐06, and NF1‐09 (related to Fig. 3).
**Fig. S6.** Phenotypic and functional characterization of established control cell lines (related to Fig. 4).
**Fig. S7.** Comparison of the main phenotypic and functional features of the two cell lines derived from SP‐01, one from the primary tumor and the other from the PDOX tumor (SP‐01‐0T).
**Table S1.** Clinical data from patients and preclinical models obtained.
**Table S2.** Summary of the genomic analyses performed in primary tumors, PDOX models, and cell lines.
**Table S3.** Short tandem repeat (STR) cell line authentication from new established cell lines.

## Data Availability

The data that support the findings of this study are available from the corresponding author (clazaro@iconcologia.net) upon reasonable request. The methylome classifier data are available through a public web link (https://doi.org/10.7303/syn52387995).
