# Supplementary material for: Expanding a precision medicine platform for malignant peripheral nerve sheath tumors: New patient‐derived orthotopic xenografts, cell lines and tumor entities
Source: Mol Oncol. 2023 Oct 20;18(4):895–917. doi: 10.1002/1878-0261.13534 (PMC10994238; doi:10.1002/1878-0261.13534)
Supplement: Supplementary file 1 — Fig. S1. Histological stains of Ki‐67, CD34, and Vimentin in the primary tumors and models. Fig. S2. LMNA‐NTRK1 gene fusion in the SP‐05 tumor. Fig. S3. Hematoxylin–Eosin (H&E) stains of primary tumors and tumors from PDOX mice. Fig. S4. Tumor‐associated fibroblasts derived from three primary tumors. Fig. S5. Copy number profile of primary tumor, orthoxenograft (PDOX) tumor, and cell line from tumors SP‐01, SP‐04, SP‐05, SP‐06, and NF1‐09 (related to Fig. 3). Fig. S6. Phenotypic and functional characterization of established control cell lines (related to Fig. 4). Fig. S7. Comparison of the main phenotypic and functional features of the two cell lines derived from SP‐01, one from the primary tumor and the other from the PDOX tumor (SP‐01‐0T). Table S1. Clinical data from patients and preclinical models obtained. Table S2. Summary of the genomic analyses performed in primary tumors, PDOX models, and cell lines. Table S3. Short tandem repeat (STR) cell line authentication from new established cell lines. [file MOL2-18-895-s001.pdf]

Supplementary Figures and Tables

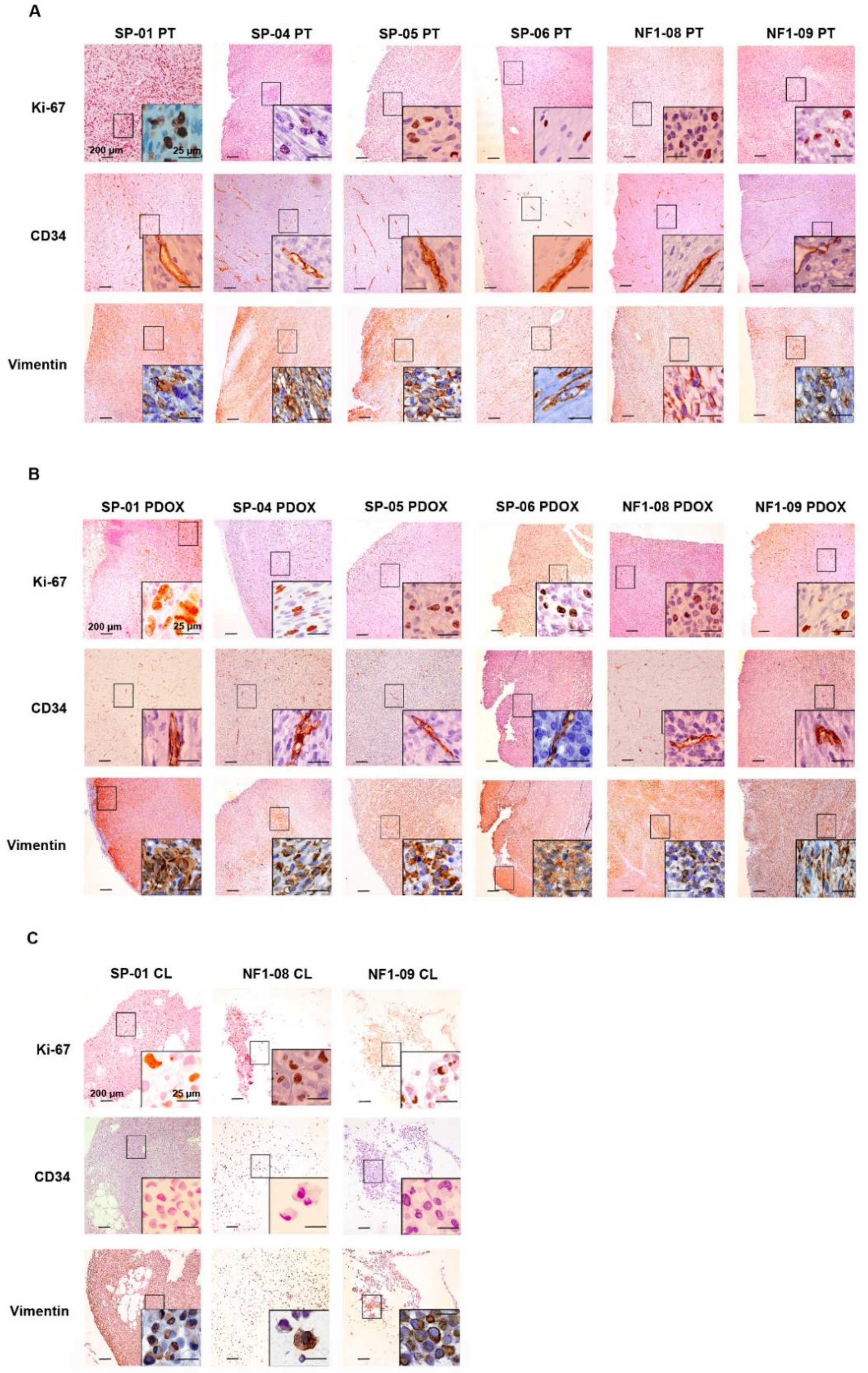

Supplementary Figure 1. Histological stains of Ki-67, CD34, and Vimentin in the primary tumors and models. A) Representative images of marker expression in primary tumors. **B)** Representative images of marker expression in PDOX tumors. **C)** Representative images of marker expression in cell lines. PT: Primary tumor. CL: Cell line. Original magnification is 40x and 600x for the inset magnified square. Scale bars are 200 µm and 25 µm.

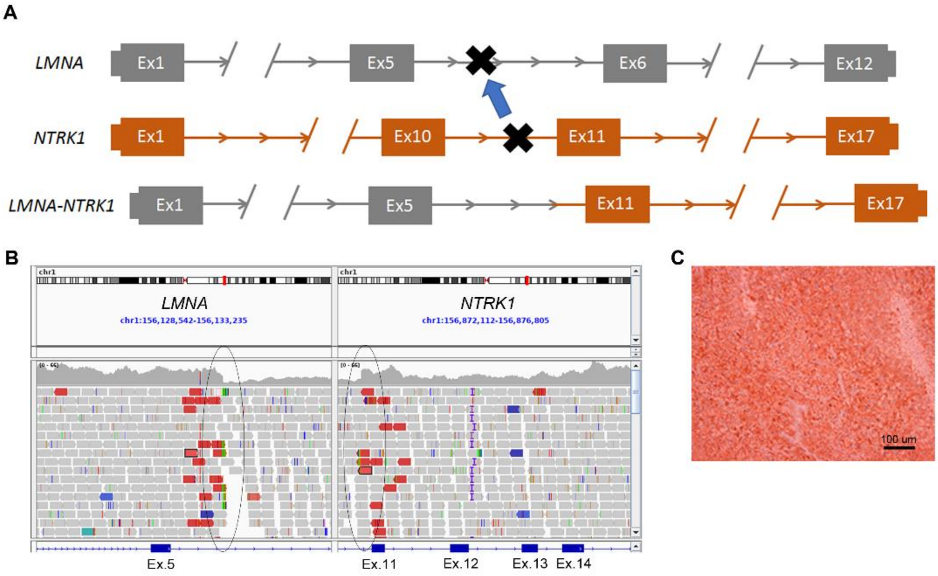

Supplementary Figure 2. *LMNA-NTRK1* gene fusion in the SP-05 tumor. **A)** Scheme of the *LMNA-NTRK1* gene fusion*.* The fusion includes the first five exons of *LMNA* and the last seven exons of *NTRK1*. **B)** Integrative Genome Viewer visualization of *LMNA* and *NTRK1* gene fusion*.* **C)** Representative image of pan-NTRK stain, which is positive and diffuse in the SP-05 tumor due to the gene fusion. Original magnification is 40x. Scale bar is 100 µm.

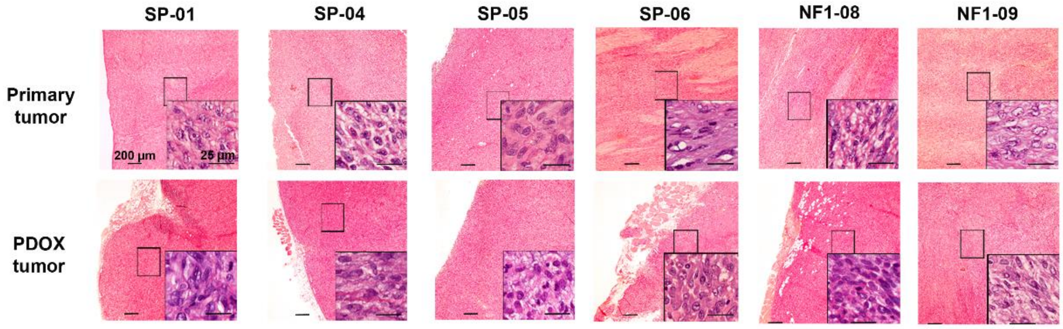

Supplementary Figure 3. Hematoxylin-Eosin (H&E) stains of primary tumors and tumors from PDOX mice. Representative images of H&E at original magnification of 40x and 600x in the magnified inset square. Scale bars are 200 µm and 25 µm.

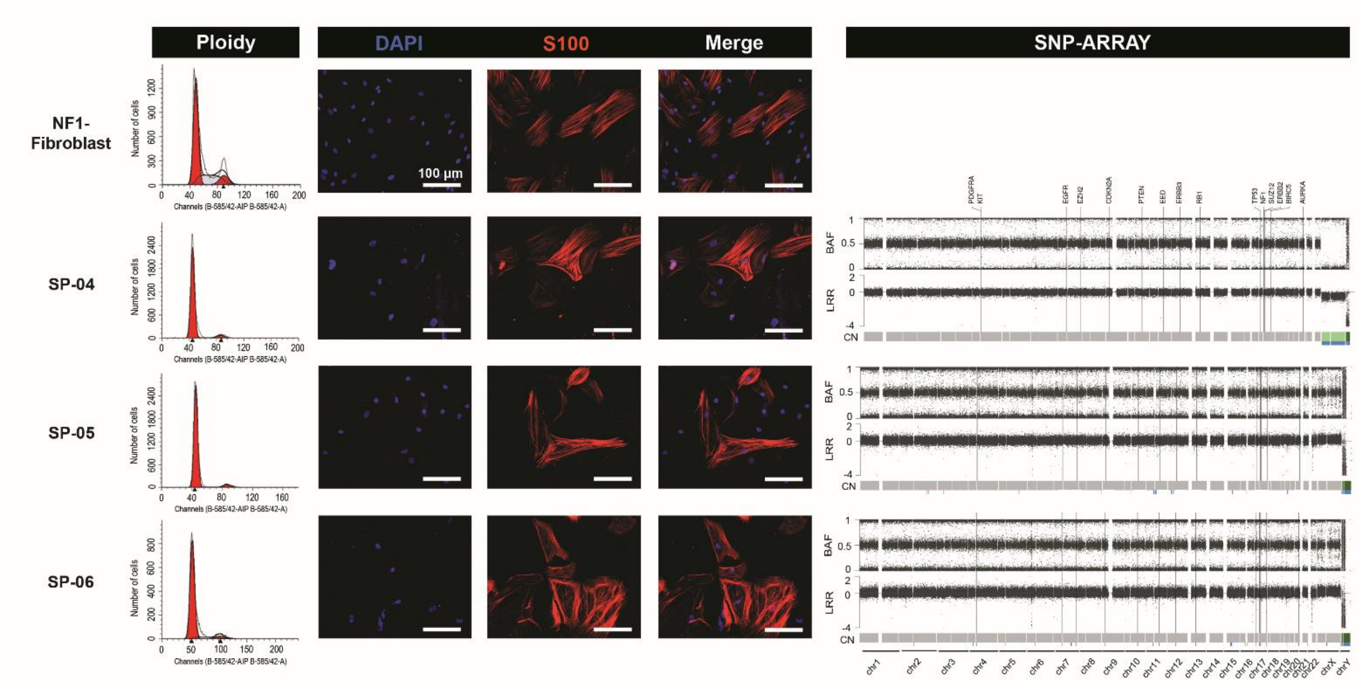

Supplementary Figure 4. Tumor-associated fibroblasts derived from three primary tumors. On the left, ploidy analyses are represented as the number of cells versus DNA quantity. Central panels show representative images (400x magnification) of SMA-positive immunofluorescence staining (red). The scale Bar is 100 µm. On the right, copy number profile obtained from SNP-array of the fibroblast cell lines. B-allele frequency (BAF) and Log-R ratio (LRR) profiles are represented.

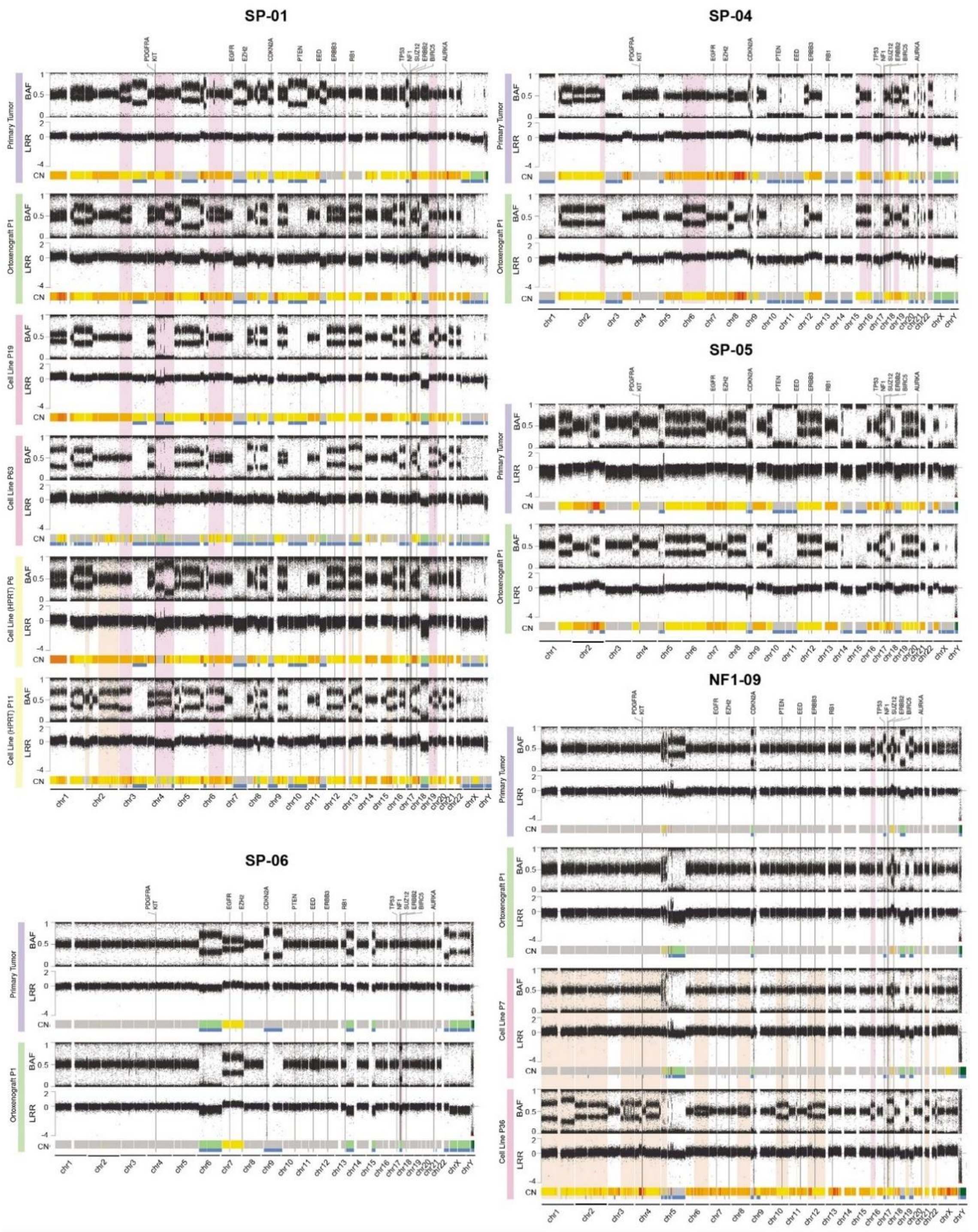

Supplementary Figure 5. Copy number profile of primary tumor, orthoxenograft (PDOX) tumor, and cell line from tumors SP-01, SP-04, SP-05, SP-06, and NF1-09 (related to Figure 3). The B-allelic frequency (BAF) and Log-R ratio (LRR) profiles are represented. Copy number (CN) variations are represented by a colored line under each LRR: gray for 2n region; yellow to red for > 2n, representing chromosomal gain; and green for < 2n, representing chromosomal loss. LOH events are shown in blue. Genomic differences between primary and xenograft tumors are highlighted in purple and differences between cell lines at low and high passage are marked in a cream color.

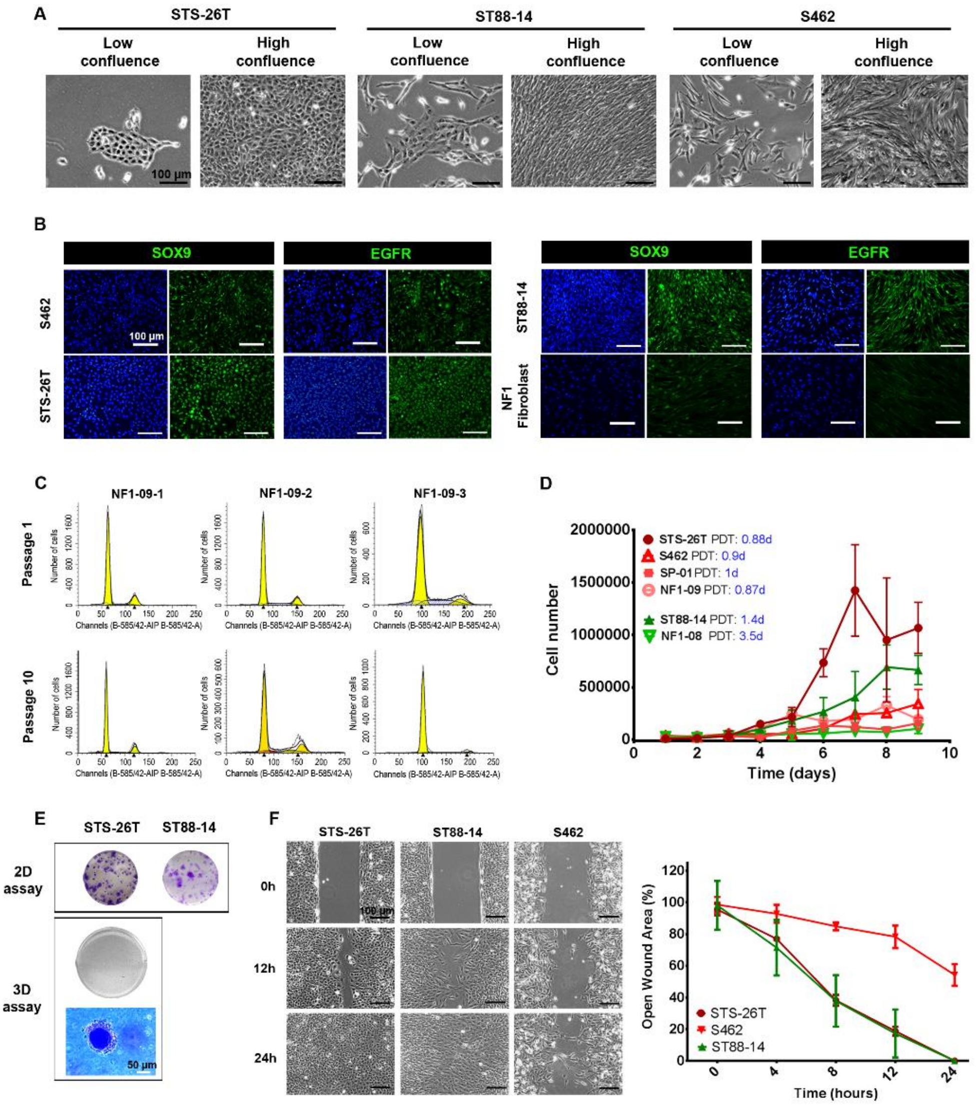

Supplementary Figure 6. Phenotypic and functional characterization of established control cell lines (related to Figure 4). A) Representative images of the cell lines morphology at low and high confluence. Original magnification is 100x and the scale bar is 100 µm. **B)** Representative images of immunofluorescence of SOX9 and EGFR markers (200x magnification)**.** The scale bar is 100 µm. **C)** DNA content analyses of the different NF1-09 cell line subpopulations, represented as number of cells versus DNA quantity, at low (passage 1) and high passage (passage 10). NF1-09-1 is between 2n and 3n, NF1-09-2 is triploid, and NF1-09-3 is tetraploid (n=2). **D)** Cell growth curves and PDT calculation using the Trypan Blue stain of the newly generated cell lines and the control established cell lines. Growth curves are derived from mean values ± SD (error bars, n=3). In red, cell lines that generate tumors in mice and, in green, cell lines that do not generate tumors. **E)** Colony formation assays of established cell lines STS-26T and ST88-14 (only in 2D). The STS-26T cell line was only cultured seven days due to high proliferation rate. Representative images were taken at 400x magnification and the scale bar is 50 µm. **F)** Wound healing assay of established control cell lines. Representative images were captured at 0, 12, and 24 h (left) (100x magnification). The migration ability of cells was represented as the percentage of open wound at 0, 4, 8, 12, and 24 h (right). Open wound curves are derived from mean values ± SD (error bars, n=3). In red, cell lines that generate tumors in mice, and in green, cell lines that do not generate tumors. The scale bar is 100 µm.

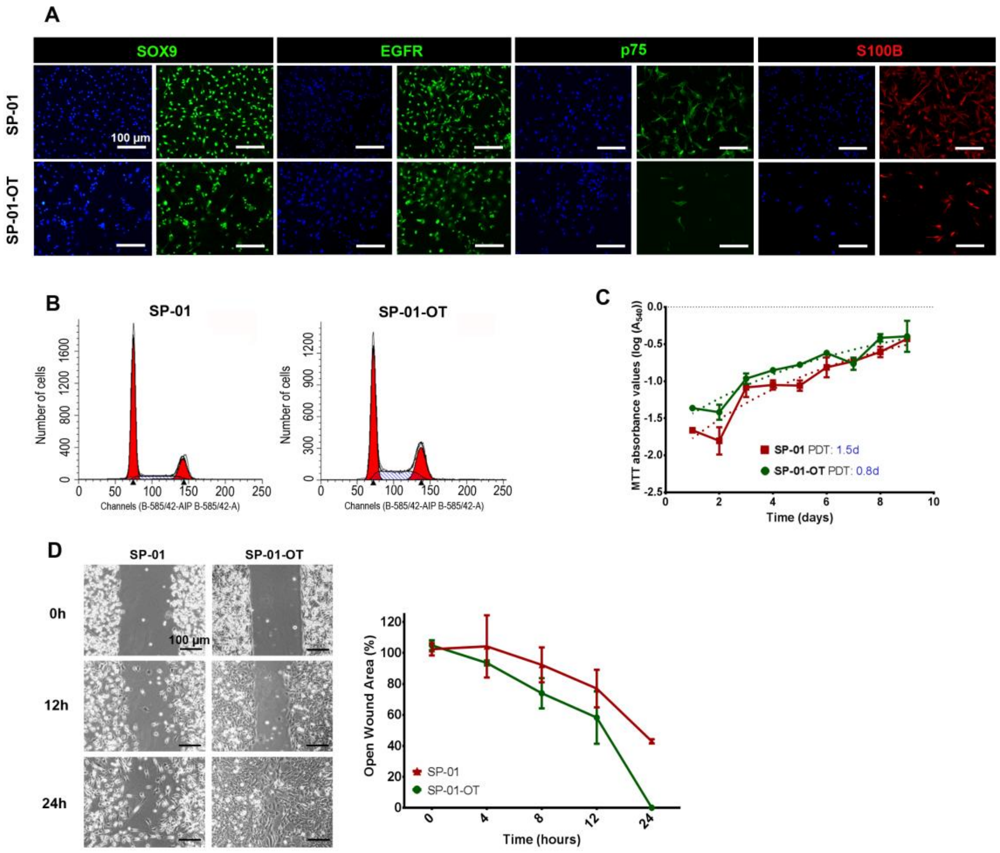

Supplementary Figure 7. Comparison of the main phenotypic and functional features of the two cell lines derived from SP-01, one from the primary tumor and the other from the PDOX tumor (SP-01-OT). A) Representative images of immunofluorescence of SOX9 and EGFR, and neural crest-Schwann cell lineage markers p75 and S100B**.** Slightly lower expression was observed in the SP-01-OT cell line. Original magnification is 200x and the scale bar is 100 µm. **B)** DNA content analyses represented as number of cells versus DNA quantity. Both cell lines presented the same degree of aneuploidy (n=2). **C)** Cell growth curves and PDT calculation using the MTT viability assay. Growth curves are derived from mean values ± SD (error bars, n=6). SP-01-OT had a lower PDT value. **D)** Wound healing assay of the two cell lines. Representative images of wound closing were captured at 0, 12, and 24 h (left) (100x magnification). The migration ability of cells was represented as the percentage of open wound at 0, 4, 8, 12, and 24 h (right). Open wound curves are derived from mean values ± SD (error bars, n=3). SP-01-OT had higher migration ability. The scale bar is 100 µm. All data and images used in this figure regarding the SP-01 cell line come from Figure 4.

Supplementary Table 1. Clinical data from patients and preclinical models obtained.

Tumor ID	Age	Sex	NF1/Sporadic	*NF1* constitutional variant	MPNST type	Tumor grade	Location	Preclinical models		
								PDOX	Cell line	CAF cell line
**SP-01***	88	M	Sporadic	NA	Primary	IV	Laterocervical	Yes	Yes	No
**SP-04**	73	M	Sporadic	NA	Primary	III	Thigh	Yes	No	Yes
**SP-05**	42	F	Sporadic	NA	Primary	High grade	Wrist	Yes	No	Yes
**SP-06**	82	F	Sporadic	NA	Primary	II	Sciatic nerve	Yes	No	Yes
**NF1-08****	28	M	NF1	NM_000267.2:c.701_730+10del	Primary	III	Dorsal	Yes	Yes	No
**NF1-09****	35	F	NF1	NM_000267.2:c.6792C>A:p.(Tyr2264*)	Primary	III	Sciatic nerve	Yes	Yes	No

*SP-01 was previously described in Castellsagué *et al*., 2015 (as MPNST-SP-001).
**NF1-08 and NF1-09 were previously described and used in Fernández-Rodríguez *et al*., 2022 (as MPNST-NF1-08 and MPNST-NF1-09).

Supplementary Table 2. Summary of the genomic analyses performed in primary tumors, PDOX models, and cell lines.

Sample ID	Sample origin	WGS	RNA-seq	WES	SNP array
**SP-01**	PT	YES	YES	YES	YES
	CL	NO	YES	YES	YES
	PDOX	NO	NO	YES	YES
**SP-04***	PT	YES	YES	YES	YES
	CL	NA	NA	NA	NA
	PDOX	NO	NO	YES	YES
**SP-05***	PT	YES	YES	YES	YES
	CL	NA	NA	NA	NA
	PDOX	NO	NO	YES	YES
**SP-06***	PT	NO	YES	YES	YES
	CL	NA	NA	NA	NA
	PDOX	NO	NO	YES	YES
**NF1-08**	PT	YES	YES	YES	YES
	CL	NO	NO	YES	YES
	PDOX	NO	NO	YES	YES
**NF1-09**	PT	YES	YES	YES	YES
	CL	NO	NO	YES	YES
	PDOX	NO	NO	YES	YES

PT: primary tumor; CL: tumor cell line; PDOX: orthoxenograft mouse tumor; NA: Not applicable
*No tumor cell line was obtained from SP-04, SP-05, and SP-06 tumors

Supplementary Table 3. Short tandem repeat (STR) cell line authentication from new established cell lines.

		SP-01					NF1-08				NF1-09			
Microsatellite	Chr. Location	Blood	PT	OT	CL	CL OT	Blood	PT	OT	CL	Blood	PT	OT	CL
D8S1179	8	12	12	12	12	12	10,14	10,14	10,14	10,14	14,15	14,15	14,15	14,15
D21S11	21q11.2-q21	30,32.2	30,32.2	30,32.2	30,32.2	30,32.2	29,31.2	29,31.2	29,31.2	29,31.2	31, 31.2	31, 31.2	31, 31.2	31, 31.2
D7S820	7q11.21-22	8,9	8,9	9	9	9	8,12	8,12	8,12	8,12	9,11	9,11	9,11	9,11
CSF1PO	5q33.3-34	11	11	11	11	11	10,12	10,12	10,12	10,12	10,12	10,12	12	12
D3S1358	3p	16,17	16,17	16,17	16,17	16,17	18	18	18	18	16,18	16,18	16,18	16,18
TH01	11p15.5	7,9.3	7,9.3	7,9.3	7,9.3	7,9.3	9.3	9.3	9.3	9.3	7,8	7,8	7,8	7,8
D13S317	13q22-31	11	11	11	11	11	11,13	11,13	11,13	11,13	11	11	11	11
D16S539	16q24-qter	12	12	12	12	12	11,12	12	12,13	12	12,13	12,13	12,13	12,13
D2S1338	2q35-37.1	16,25	16,25	16,25	16,25	16,25	19,23	19,23	19,23	19,23	17,23	17,23	17,23	17,23
D19S433	19q12-13.1	12	12	12	12	12	15, 16.2	15, 16.2	15, 16.2	15, 16.2	14, 15.2	14, 15.2	15.2	15.2
vWA	12p12-pter	14,16	14,16	14,16	14,16	14,16	16,18	16	16	16	14,18	14,18	14,18	14,18
TPOX	2p23-2per	8,11	8,11	8,11	8,11	8,11	8,11	8,11	8,11	8,11	8,9	8,9	8,9	8,9
D18S51	18q21.3	12,14	12,14	12,14	14	14	15,17	15,18	15,18	15,18	14,15	14,15	14	14
AMEL	Xp22.1 Yp11.2	X,Y	X,Y	X,Y	X,Y	X,Y	X,Y	X,Y	X,Y	X,Y	X	X	X	X
D5S818	5q21-31	11,12	11,12	11,12	11,12	11,12	12	12	12	12	11,12	11,12	12	12
FGA	4q28	19,25	19,25	19,25	19	19,26	22,26	26	26	26	20,21	20,21	20,21	20,21

PT: Primary Tumor; OT: Orthotopic tumor; CL: Cell line; CL OT: Cell line derived from PDOX tumor
